# Multisystem frailty phenotypes and associated factors among older adults in Türkiye: A nationally representative study

**DOI:** 10.1371/journal.pone.0352767

**Published:** 2026-06-26

**Authors:** Salim Yılmaz, Yusuf Çelik, Elif Cansu Kara

**Affiliations:** 1 Department of Healthcare Management, Faculty of Health Sciences, Acibadem Mehmet Ali Aydinlar University, Istanbul, Türkiye; 2 Department of Healthcare Management, Graduate School of Health Sciences, Acibadem Mehmet Ali Aydinlar University, Istanbul, Türkiye; Instituto Nacional de Geriatria, MEXICO

## Abstract

Multisystem frailty, characterised by concurrent decline across motor, sensory, cognitive, and functional domains, remains a relatively understudied phenotype within ageing research. Evidence on its population-level distribution and associated factors, particularly in middle-income countries, is scarce. This study aimed to identify distinct multisystem frailty profiles among adults aged 50 years and older in Türkiye using nationally representative data. We analysed data from 26,905 adults aged 50 years and older drawn from the 2023 Turkish Statistical Institute Elderly Statistics Survey. Latent profile analysis was employed using eight indicators capturing motor, sensory, cognitive, and functional domains. Survey-weighted multinomial logistic regression models were used to examine sociodemographic and health-related factors associated with frailty profile membership. Three multisystem frailty profiles were identified: robust (weighted prevalence 51.4%), intermediate frailty (33.7%), and severe multisystem frailty (14.8%). Severe frailty was substantially more common among women than men (20.0% vs 9.1%) and among adults aged 65 years and older compared with those aged 50–64 years (24.7% vs 8.9%). Health- and lifestyle-related factors accounted for the largest share of explained variance in profile membership (51.9%). Poor or very poor self-rated health exhibited exceptionally strong associations with severe frailty (odds ratio range 47.89–95.78), with corresponding E-values exceeding 90. Multisystem frailty affects nearly half of adults aged 50 years and older in Türkiye and displays pronounced inequalities by age, sex, and perceived health status. These findings underscore the importance of multidomain assessment approaches and highlight potentially modifiable social and health-related factors associated with frailty vulnerability in ageing populations.

## Introduction

Population ageing represents one of the most significant demographic shifts of the 21st century. Globally, the number of persons aged 65 years and older is projected to reach 2.2 billion by the late 2070s, more than doubling from current levels [1]. Frailty, a state of increased vulnerability to adverse health outcomes resulting from age-related decline across multiple physiological systems, has emerged as a central concept in geriatric medicine [2]. Unlike chronological age alone, frailty captures the heterogeneity of the ageing process and identifies individuals at heightened risk of adverse outcomes. A recent meta-analysis of 56 prospective studies involving over 1.8 million community-dwelling adults demonstrated that frail individuals face a 2.4–fold higher risk of all-cause mortality (HR 2.40, 95% CI 2.17–2.65) compared with their robust counterparts [3]. As populations age and health systems face mounting pressure, accurate identification and characterisation of frailty phenotypes has become a policy priority. Within this context, frailty has emerged as a key construct capturing vulnerability across multiple physiological systems in later life.

The most widely used definition of frailty, proposed by Fried and colleagues [4], conceptualises the syndrome as a physical phenotype characterised by five criteria: unintentional weight loss, exhaustion, weakness, slow walking speed, and low physical activity. Although this phenotypic approach has demonstrated strong predictive validity for adverse outcomes, it primarily captures a single dimension of the ageing process. Growing evidence indicates that frailty extends beyond physical decline to encompass sensory, cognitive, and functional domains [5]. Motor impairments, such as gait instability, frequently co-occur with sensory deficits in vision and hearing, cognitive changes affecting memory and attention, and functional limitations in activities of daily living. This clustering of impairments across domains suggests that frailty reflects a shared underlying vulnerability rather than a set of independent, organ-specific deficits. Such a multisystem perspective is consistent with contemporary views of ageing as a process of cumulative multisystem vulnerability rather than isolated physical deterioration.

Despite increasing recognition of frailty’s multidimensional nature, population-based evidence from middle-income countries remains limited [6,7]. Türkiye exemplifies this gap: individuals aged 65 years and older currently account for 10.6% of the population (9.1 million persons) and are projected to reach 17.9% by 2040, highlighting the growing need for scalable and context-appropriate frailty assessment strategies [8]. While previous studies in Türkiye have examined frailty using modified Fried criteria in regional or subnational samples [9], no nationally representative study has characterised frailty phenotypes that integrate motor, sensory, cognitive, and functional indicators.

This study aimed to address these gaps by using nationally representative data from the 2023 Elderly Statistics Survey. We aimed to characterise frailty phenotypes by applying latent profile analysis to eight indicators spanning motor, sensory, cognitive, and functional domains, thereby conceptualising frailty as a multisystem construct in a data-driven manner. Beyond estimating the prevalence of frailty profiles, we examined the relative and unique contributions of demographic, socioeconomic, health-related, and lifestyle factors through a hierarchical modelling approach. This variance decomposition framework offers policy-relevant insights by distinguishing factors that show independent associations with frailty from those whose associations are substantially attenuated after adjustment for other covariates. Finally, the robustness of key associations to potential unmeasured confounding was evaluated using E-value sensitivity analyses.

## Methods

### Study design

This study is a secondary analysis of cross-sectional data obtained from the 2023 Turkish Statistical Institute (TURKSTAT) Elderly Statistics Survey, a nationally representative household survey of older adults in Türkiye. The survey employs a stratified two-stage cluster sampling design covering the country’s 12 NUTS–1 regions [8]. Data were collected through face-to-face interviews with individuals aged 50 years and older residing in private households.

**Fig 1:**
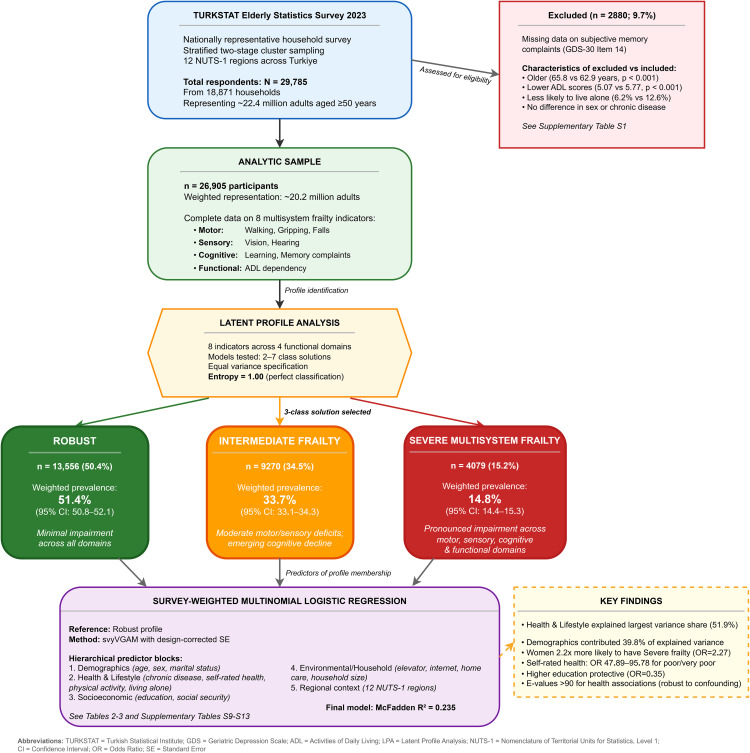
Study flow diagram and multisystem frailty profile classification.

The analytic sample comprised 29 785 individuals from 18 871 households; when survey weights were applied, the data represented approximately 22.4 million persons aged 50 years and older nationwide. All analyses accounted for the complex survey design, including sampling weights, strata, and primary sampling units (Fig 1).

### Participants

The original survey sample consisted of 29,785 individuals (Fig 1). Participants with missing data on the latent profile analysis indicator variables were excluded (n = 2880; 9.7%), and the analyses were carried out with the remaining 26,905 individuals. Missing data were confined to the subjective memory complaints item derived from the Geriatric Depression Scale (GDS); all other multisystem frailty indicators were complete. Missing data patterns and comparisons between included and excluded participants are presented in Supplementary Material S1 in S1 File.

### Measures and survey weighting

Multisystem frailty was estimated by using eight indicators spanning four domains: motor, sensory, cognitive, and functional. Motor function was assessed through three indicators: difficulty in walking, difficulty in grasping objects, and self-reported falls within the past 12 months. Sensory function included difficulty in seeing and difficulty in hearing. These five indicators were measured using the Washington Group Short Set on Functioning (WG-SS), a standardised instrument endorsed by the United Nations for internationally comparable assessment of functional limitations [10]. All WG-SS items employed a four-level response scale ranging from 0 (“no difficulty”) to 3 (“cannot do at all”).

Cognitive function was captured using two indicators: difficulty in learning new tasks, derived from the WG-SS, and subjective memory complaints, assessed using Item 14 of the 30-item Geriatric Depression Scale (GDS): “Do you feel you have more problems with memory than most people?” [11]. The Turkish version of the GDS has demonstrated adequate psychometric validity and reliability [12].

Functional dependence was measured using the Katz Index of Activities of Daily Living (ADL), which assesses independence in bathing, dressing, toileting, transferring, continence, and feeding. Dependency was defined as the presence of any limitation, corresponding to a total score of less than six [13].

Covariates included a comprehensive set of sociodemographic, socioeconomic, health-related, and environmental characteristics, as detailed in Supplementary Material S2 in S1 File. All descriptive statistics and regression analyses incorporated individual-level sampling weights to account for the complex survey design and to produce nationally representative estimates.

### Statistical analysis

All analyses were conducted by using R version 4.4.2 (Supplementary Material S13 in S1 File). Descriptive statistics were weighted by using individual-level survey weights and stratified by sex and age group (50–64 vs ≥ 65 years). Group differences were assessed by using design-based Rao–Scott χ² tests for categorical variables and Wald tests for continuous variables.

Prior to latent profile analysis (LPA) [14], polychoric correlations among the eight multisystem frailty indicators were examined to assess potential multicollinearity (S1 Fig). LPA models specifying between two and seven classes were estimated using the tidyLPA package under two parameterisations: equal versus freely varying within-class variances, with covariances constrained to zero in both cases, and 100 random starts [15]. Model selection was guided by multiple criteria, including the Akaike Information Criterion (AIC), Bayesian Information Criterion (BIC), sample-size adjusted BIC (ssaBIC), bootstrap likelihood ratio test (BLRT), entropy, minimum class size (≥3%), and substantive interpretability. Classification quality was further evaluated by using average posterior probabilities (Supplementary Table S4 in S1 File).

As tidyLPA does not accommodate survey weights, unweighted models were used for class enumeration. Survey-weighted prevalence estimates and corresponding 95% confidence intervals were subsequently calculated overall and stratified by sex and age group. Structural consistency of the selected profile solution was assessed by estimating separate LPA models within key demographic subgroups (sex and age groups) and comparing class convergence, entropy, and indicator response patterns (Supplementary Table S8 in S1 File).

Associations between covariates and frailty profile membership were examined by using survey-weighted multinomial logistic regression with design-corrected standard errors implemented via the svyVGAM package [16]. Prior to regression modelling, intraclass correlation coefficients (ICCs) were estimated at the household and regional levels to assess clustering. Both ICCs were negligible (<0.05), supporting the use of single-level survey-weighted models rather than multilevel approaches [17] (Supplementary Table S11 in S1 File).

The regression models included demographic (age, sex, marital status), socioeconomic (education, social security coverage, living alone, household size), health-related (chronic disease, self-rated health, physical activity), and environmental factors (elevator access, internet access, home care need) as covariates, with the Robust profile specified as the reference category. Wald tests were used to assess the joint significance of each predictor across the two non-reference outcome categories (Intermediate frailty and Severe frailty). Model fit was evaluated by using McFadden’s pseudo-R^2^. To quantify the relative contribution of predictor domains, hierarchical regression models were estimated by sequentially introducing covariate blocks in a theoretically informed order, with incremental pseudo-R^2^ calculated for each block.

Stratified analyses by sex and age group were conducted to explore potential effect modification (Supplementary Tables S9–S10 in S1 File). To contextualise the LPA-derived profiles against conventional frailty frameworks, two additional classifications were constructed from the available survey data. A modified Fried phenotype was operationalised using three proxy criteria approximating the original Fried criteria [4]: substantial walking difficulty (Washington Group score ≥2) as a proxy for slowness, substantial gripping difficulty (score ≥2) as a proxy for weakness, and no leisure-time physical activity as a proxy for low activity. Participants meeting zero, one, or two or more criteria were classified as robust, pre-frail, or frail, respectively. Two of the five original Fried criteria—unintentional weight loss and exhaustion—were not available in the survey instrument. Additionally, a Frailty Index (FI) was constructed using the deficit accumulation approach [18], incorporating 11 health deficits rescaled to a 0–1 range, with standard cutpoints applied (FI < 0.10 robust, 0.10–0.25 pre-frail, > 0.25 frail). Cross-classification tables, domain-specific impairment profiles of discordant cases, and Cohen’s kappa agreement statistics were computed to compare classification approaches (Supplementary Material S11, Table S13 in S1 File).

Sensitivity to unmeasured confounding was assessed using E-values, which represent the minimum strength of association that an unmeasured confounder would need to have with both the exposure and outcome to fully account for the observed associations (Supplementary Table S14 in S1 File) [19,20]. All statistical tests were two-sided, and p values <0.05 were considered statistically significant.

### Patient consent / ethics

This study is a secondary analysis of fully anonymised microdata provided by the Turkish Statistical Institute (TURKSTAT), the official national statistical authority of the Republic of Türkiye. The data are not publicly available and were accessed under a formal data-use agreement for research purposes only. The dataset contains no personally identifiable information, and individual participants cannot be identified. In accordance with national regulations and institutional guidelines, analyses based exclusively on anonymised secondary data do not require approval from an institutional ethics committee. Permission to access and use the data was granted by TURKSTAT, and all analyses complied with applicable data protection and confidentiality requirements.

## Results

The weighted sample represented approximately 20.2 million adults aged 50 years and older in Türkiye, of whom 47.3% were men and 52.7% were women (Table 1). The median age was 61 years (IQR 55–69). Women were marginally older than men (median 62 vs 61 years; mean 63.1 vs 62.2 years, p < 0.001) and were more likely to belong to the 65 years and older age group (39.4% vs 35.7%).

**Table 1:** Sample characteristics by sex (survey-weighted).

Characteristic	Overall *(N = 20,174,792)*	Men *(n = 9 549,321)*	Women *(n = 10,625,471)*	p-value
**Age, years, mean (SD)**	62.7 (9.4)	62.2 (9.1)	63.1 (9.7)	<0.001
**Age group, n (%)**				
50-64 years	12,570,708 (62.3)	6,136,909 (64.3)	6,433,799 (60.6)	<0.001
65 + years	7,604,084 (37.7)	3,412,412 (35.7)	4,191,672 (39.4)	
**Marital status, n (%)**				
Married	15,410,136 (76.4)	8,377,059 (87.7)	7,033,078 (66.2)	<0.001
Widowed	3,261,157 (16.2)	523,845 (5.5)	2,737,311 (25.8)	
Divorced	1,021,687 (5.1)	451,354 (4.7)	570,333 (5.4)	
Never married	476,009 (2.4)	197,063 (2.1)	278,946 (2.6)	
**Education, n (%)**				
No formal education	3,760,103 (18.6)	639,019 (6.7)	3,121,084 (29.4)	<0.001
Primary school	10,026,797 (49.7)	4,933,980 (51.7)	5,092,817 (47.9)	
Secondary school	1,637,922 (8.1)	1,052,977 (11.0)	584,945 (5.5)	
High school	2,501,646 (12.4)	1,497,117 (15.7)	1,004,528 (9.5)	
Higher education	2,248,324 (11.1)	1,426,228 (14.9)	822,096 (7.7)	
**Living alone, n (%)**				
Yes	2,640,235 (13.1)	849,582 (8.9)	1,790,654 (16.9)	<0.001
**Household size, mean (SD)**	3.1 (1.8)	3.2 (1.8)	2.9 (1.8)	<0.001
**Social security coverage, n (%)**				
Yes	17,625,106 (87.4)	8,543,282 (89.5)	9,081,823 (85.5)	<0.001
**Chronic disease, n (%)**				
Yes	12,892,062 (63.9)	5,355,237 (56.1)	7,536,825 (70.9)	<0.001
**Self-rated health, n (%)**				
Very good	511,434 (2.5)	333,139 (3.5)	178,296 (1.7)	<0.001
Good	6,609,708 (32.8)	3,714,075 (38.9)	2,895,634 (27.3)	
Fair	9,425,679 (46.7)	4,153,198 (43.5)	5,272,481 (49.6)	
Poor	3,267,051 (16.2)	1,227,520 (12.9)	2,039,531 (19.2)	
Very poor	360,919 (1.8)	121,390 (1.3)	239,529 (2.3)	
**Tobacco use, n (%)**				
Daily	4,406,099 (21.8)	3,238,586 (33.9)	1,167,513 (11.0)	<0.001
Occasional	600,712 (3.0)	340,666 (3.6)	260,047 (2.4)	
Former	3,481,390 (17.3)	2,734,045 (28.6)	747,345 (7.0)	
Never	11,686,591 (57.9)	3,236,025 (33.9)	8,450,566 (79.5)	
**Physical activity, n (%)**				
Daily or almost daily	4,451,972 (22.1)	2,617,077 (27.4)	1,834,895 (17.3)	<0.001
At least weekly	2,525,503 (12.5)	1,147,251 (12.0)	1,378,251 (13.0)	
1–3 times monthly	857,609 (4.3)	416,466 (4.4)	441,143 (4.2)	
Rarely	3,800,466 (18.8)	1,777,885 (18.6)	2,022,581 (19.0)	
Never	8,539,243 (42.3)	3,590,642 (37.6)	4,948,601 (46.6)	
**Elevator access, n (%)**				
Yes	5,269,578 (26.1)	2,498,036 (26.2)	2,771,542 (26.1)	0.900
**Internet access, n (%)**				
Yes	11,478,145 (56.9)	5,700,093 (59.7)	5,778,053 (54.4)	<0.001
**Home care need, n (%)**				
Yes	1,811,490 (9.0)	656,080 (6.9)	1,155,410 (10.9)	<0.001

Data are weighted n (%) or mean (SD). p-values from design-based Rao-Scott chi-square test for categorical variables and Wald test for continuous variables.

Substantial sex differences were observed across socioeconomic and health-related characteristics. Educational attainment was significantly lower among women, with 29.4% reporting no formal education compared with 6.7% of men, whereas men were approximately twice as likely to have completed higher education (14.9% vs 7.7%; p < 0.001). Women were also more frequently widowed (25.8% vs 5.5%), more likely to live alone (16.9% vs 8.9%), and more likely to report the presence of at least one chronic condition (70.9% vs 56.1%; all p < 0.001). Median household size was smaller among women than men (2 vs 3 persons). Although social security coverage was high overall (87.4%), it remained significantly lower among women compared with men (85.5% vs 89.5%; p < 0.001).

Health behaviours and perceived health also differed by sex. Women were more likely to report poor or very poor self-rated health (21.5% vs 14.2%), whereas men reported substantially higher rates of daily tobacco use (33.9% vs 11.0%) and more frequent engagement in physical activity (daily or almost daily: 27.4% vs 17.3%). Women additionally reported a greater need for home-based care (10.9% vs 6.9%; p < 0.001). No statistically significant sex difference was observed in access to an elevator in the residential building (p = 0.9).

Age-stratified comparisons revealed clear gradients in health and living circumstances. Adults aged 65 years and older had a substantially higher prevalence of chronic disease (78.3% vs 55.2%), were more likely to live alone (21.5% vs 8.0%), and more frequently reported a need for home-based care (14.6% vs 5.6%) than those aged 50–64 years (Supplementary Material S3 in S1 File).

**Fig 2:**
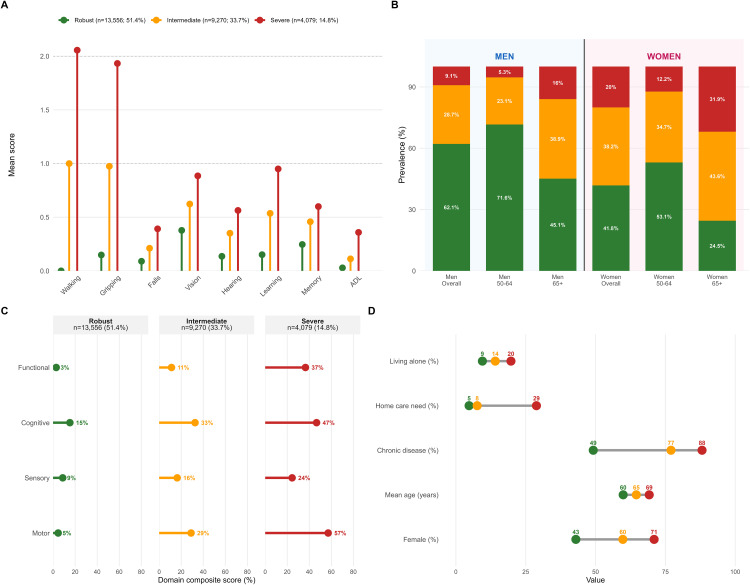
Characteristics of the three multisystem frailty profiles among Turkish adults aged 50 years and older. (A) Mean indicator scores across the three latent profiles. Scores range from 0 (no difficulty) to 3 (cannot do at all) for Washington Group indicators; falls, memory complaints, and ADL dependency are binary (0–1). (B) Survey-weighted prevalence of profiles by sex and age group, illustrating the compounding effects of female sex and older age on frailty risk. (C) Domain composite scores by profile, calculated as normalized means across indicators within each domain: Motor (walking difficulty, gripping difficulty, falls), Sensory (vision difficulty, hearing difficulty), Cognitive (learning difficulty, memory complaints), and Functional (ADL dependency). (D) Demographic and clinical characteristics by profile, showing the gradient in female proportion, mean age, chronic disease prevalence, home care need, and living alone status across frailty severity. Sample sizes and percentages in panels A and C are unweighted; prevalence estimates in panel B are survey-weighted. ADL: Activities of Daily Living; LPA model entropy = 1.00.

Latent profile analysis identified three distinct multisystem frailty profiles (Fig 2). Based on survey-weighted estimates, the Robust profile was the most prevalent, encompassing approximately 10.4 million individuals (51.4%, 95% CI 50.8–52.1). This profile was characterised by minimal impairment across all domains, including near-zero motor difficulty, low levels of sensory and cognitive complaints, and a low prevalence of ADL dependency (3%). The intermediate frailty profile included an estimated 6.8 million individuals (33.7%, 95% CI 33.1–34.3) and was marked by moderate motor impairment (mean walking difficulty 1.0), elevated sensory limitations, and an ADL dependency prevalence of 11%. The severe frailty profile, affecting approximately 3.0 million individuals (14.8%, 95% CI 14.4–15.3), exhibited substantial impairment across all assessed domains, including pronounced motor limitations (mean walking difficulty 2.1), the highest prevalence of falls (39%), and a markedly elevated prevalence of ADL dependency (37%).

Model fit indices strongly supported the three-profile solution. Compared with the two-profile model, the selected solution demonstrated a substantially improved fit (ΔBIC = 63,328) and near-perfect classification accuracy (entropy = 1.00). Models specifying more than three profiles failed to converge and were therefore not retained for further consideration (Supplementary Material S5 in S1 File).

Significant sociodemographic differences were observed in the distribution of multisystem frailty profiles (Fig 2B). Women were considerably less likely than men to be classified as Robust (41.8% vs 62.1%) and more than twice as likely to belong to the Severe frailty profile (20.0% vs 9.1%; p < 0.001). Similarly, adults aged 65 years and older exhibited a substantially higher prevalence of Severe frailty compared with those aged 50–64 years (24.7% vs 8.9%; p < 0.001) and were markedly less likely to be classified as Robust (33.8% vs 62.1%).

Joint stratification by sex and age revealed a pronounced gradient in frailty burden. Men aged 50–64 years displayed the lowest prevalence of Severe frailty (5.3%) and the highest prevalence of Robust status (71.6%). In contrast, women aged 65 years and older exhibited the highest prevalence of Severe frailty (31.9%) and the lowest prevalence of Robust status (24.5%) (Supplementary Material S6 in S1 File).

Profile characteristics exhibited clear and monotonic gradients across increasing frailty severity. Individuals classified within the Robust profile showed minimal motor impairment (mean walking difficulty 0.00; mean gripping difficulty 0.15), low levels of sensory and cognitive complaints, and a very low prevalence of ADL dependency (3%). The Intermediate frailty profile was characterised by moderate multisystem impairment, including elevated walking (mean 1.00) and gripping difficulties (mean 0.98), and an ADL dependency prevalence of 11%. In contrast, the Severe multisystem frailty profile demonstrated substantial deficits across all assessed domains, with pronounced walking (mean 2.06) and gripping impairments (mean 1.92), and a high prevalence of ADL dependency (37%).

Composite domain scores confirmed a consistent severity gradient across profiles, with motor impairment displaying the steepest increase, rising from 5% in the Robust profile to 57% in the Severe profile (Fig 2C). Demographic and health characteristics further differentiated the profiles: compared with individuals in the Robust profile, those in the Severe profile were older (mean age 69 vs 60 years), more likely to be women (71% vs 43%), and had markedly higher prevalence of chronic disease (88% vs 49%) and home care needs (29% vs 5%) (Fig 2D). The near-perfect entropy value (1.00) indicates a high degree of certainty in the classification of individuals into distinct multisystem frailty phenotypes. Subgroup analyses demonstrated that the three-profile structure was replicated among women and adults aged 65 years and older, whereas among men only a two-profile solution converged (Supplementary Material S8 in S1 File).

Comparison with conventional frailty classifications revealed notable differences in prevalence and case identification. The modified Fried phenotype yielded a similar overall frailty prevalence (49.2%) to the LPA multisystem approach (48.6%), whereas the Frailty Index produced a substantially higher estimate (87.9%), reflecting the inclusion of highly prevalent deficits such as chronic disease and suboptimal self-rated health. Cross-classification of LPA profiles against the modified Fried phenotype indicated high concordance for the Severe profile (93.2% classified as Fried frail) but substantial discordance for the Intermediate profile, in which 47.9% of individuals (n = 4,441) were classified as robust by the modified Fried criteria despite exhibiting multisystem impairment. These discordant cases—constituting 16.5% of the total analytic sample—were characterised by sensory and cognitive vulnerability (mean vision difficulty 0.59; 42.1% memory complaints) with motor impairment below the modified Fried threshold. Only 96 individuals (0.4%) were classified as frail by the modified Fried criteria but robust by LPA, indicating that the multisystem approach captured nearly all individuals identified by the physical phenotype plus a substantial additional subpopulation characterised by non-motor impairment (Supplementary Material S11, Table S13 in S1 File).

Survey-weighted multinomial logistic regression analyses identified several independent predictors of multisystem frailty profile membership. All covariates, with the exception of living alone, exhibited statistically significant joint associations with frailty profile membership across outcome categories (Table 2).

**Table 2:** Survey-weighted multinomial logistic regression for predictors of multisystem frailty profiles.

Variable	Intermediate vs Robust		Severe vs Robust		p (overall)
	OR (95% CI)	p-value	OR (95% CI)	p-value	
Demographics					
**Age** (per year)	1.05 (1.04–1.05)	<0.001	1.08 (1.07–1.09)	<0.001	<0.001
**Sex** (Women vs *Men*)	1.59 (1.48–1.71)	<0.001	2.25 (2.02–2.51)	<0.001	<0.001
**Marital status** *(ref: Divorced)*					
Widowed	1.24 (1.04–1.47)	0.019	1.32 (1.01–1.72)	0.045	<0.001
Married	1.17 (0.99–1.39)	0.061	1.07 (0.83–1.38)	0.608	
Never married	0.77 (0.59–1.00)	0.05	0.85 (0.56–1.30)	0.452	
Socioeconomic factors					
**Education** *(ref: No formal education)*					
Primary school	0.77 (0.70–0.85)	<0.001	0.71 (0.62–0.81)	<0.001	<0.001
Secondary school	0.65 (0.56–0.75)	<0.001	0.59 (0.47–0.75)	<0.001	
High school	0.51 (0.45–0.59)	<0.001	0.35 (0.28–0.44)	<0.001	
Higher education	0.45 (0.39–0.53)	<0.001	0.34 (0.27–0.44)	<0.001	
**Social security** (No vs *Yes*)	1.26 (1.13–1.40)	<0.001	1.20 (1.04–1.39)	0.012	<0.001
Living arrangement					
Living alone (Yes vs *No*)	1.09 (0.95–1.25)	0.218	0.96 (0.79–1.15)	0.641	0.214
Household size (per person)	1.04 (1.02–1.07)	0.002	1.04 (1.00–1.08)	0.027	0.005
Health status					
**Chronic disease** (Yes vs *No*)	1.55 (1.43–1.68)	<0.001	1.47 (1.28–1.69)	<0.001	<0.001
**Self–rated health** *(ref: Very good)*					
Good	1.93 (1.42–2.63)	<0.001	1.38 (0.71–2.68)	0.347	<0.001
Fair	4.77 (3.50–6.51)	<0.001	7.14 (3.70–13.77)	<0.001	
Poor	8.80 (6.36–12.18)	<0.001	42.94 (22.11–83.36)	<0.001	
Very poor	5.70 (3.50–9.29)	<0.001	87.56 (41.68–183.95)	<0.001	
**Physical activity** *(ref: 1–3 times monthly)*					
Daily or almost daily	0.61 (0.52–0.72)	<0.001	0.49 (0.36–0.67)	<0.001	<0.001
At least weekly	0.83 (0.70–0.98)	0.028	0.78 (0.57–1.06)	0.117	
Rarely	0.95 (0.80–1.11)	0.496	1.27 (0.95–1.70)	0.100	
Never	1.12 (0.96–1.31)	0.153	2.09 (1.58–2.75)	<0.001	
Environmental factors					
Elevator access (Yes vs *No*)	0.87 (0.80–0.94)	<0.001	0.89 (0.78–1.00)	0.055	0.002
Internet access (Yes vs *No*)	0.87 (0.81–0.94)	<0.001	0.94 (0.84–1.05)	0.248	<0.001
Home care need (Yes vs *No*)	0.90 (0.79–1.03)	0.139	2.23 (1.92–2.59)	<0.001	<0.001

Results are from a single multinomial logistic regression model with three outcome categories; Robust profile serves as the reference. For binary variables, the first category is compared against the second (reference). p (overall) represents joint Wald test for the overall effect of each predictor across both outcome categories. OR: odds ratio; CI: confidence interval. Estimates derived from survey-weighted multinomial logistic regression with design-corrected standard errors. McFadden pseudo-R^2^ = 0.230. n = 26,898.

Among demographic factors, female sex was associated with substantially higher odds of both Intermediate frailty (OR 1.59, 95% CI 1.48–1.71) and Severe frailty (OR 2.25, 95% CI 2.02–2.51) compared with men. Increasing age was also strongly associated with frailty severity, with each additional year associated with higher odds of Intermediate (OR 1.05, 95% CI 1.04–1.05) and Severe frailty (OR 1.08, 95% CI 1.07–1.09). Relative to divorced individuals, widowhood was associated with higher odds of both frailty profiles, whereas never-married individuals showed non-significant trends towards lower odds.

Socioeconomic factors exhibited significant pattern. Educational attainment demonstrated a strong protective association: compared with individuals with no formal education, those with higher education had 55% lower odds of Intermediate frailty (OR 0.45, 95% CI 0.39–0.53) and 66% lower odds of Severe frailty (OR 0.34, 95% CI 0.27–0.44). Lack of social security coverage was independently associated with increased odds of both Intermediate (OR 1.26, 95% CI 1.13–1.40) and Severe frailty (OR 1.20, 95% CI 1.04–1.39), suggesting a potential link between healthcare access and frailty risk.

Health-related factors showed the strongest associations with frailty profile membership. Self-rated health emerged as the most powerful predictor: compared with very good health, poor self-rated health was associated with significantly higher odds of Intermediate (OR 8.80, 95% CI 6.36–12.18) and Severe frailty (OR 42.94, 95% CI 22.11–83.36). Notably, very poor self-rated health was associated with lower odds of Intermediate frailty (OR 5.70) but substantially higher odds of Severe frailty (OR 87.56, 95% CI 41.68–183.95), suggesting that individuals with the poorest perceived health may be disproportionately represented in the severe rather than the intermediate profile. The presence of chronic disease was also associated with increased odds of both Intermediate (OR 1.55, 95% CI 1.43–1.68) and Severe frailty (OR 1.47, 95% CI 1.28–1.69).

Physical activity demonstrated a consistent protective association. Daily or near-daily physical activity was associated with 39% lower odds of Intermediate frailty (OR 0.61, 95% CI 0.52–0.72) and 51% lower odds of Severe frailty (OR 0.49, 95% CI 0.36–0.67). In contrast, physical inactivity was associated with more than doubled odds of Severe frailty (OR 2.09, 95% CI 1.58–2.75) but showed no statistically significant association with Intermediate frailty, suggesting a stronger association with advanced impairment.

Among environmental factors, elevator access and internet access were modestly associated with lower odds of Intermediate frailty. Home care need was strongly associated with Severe frailty (OR 2.23, 95% CI 1.92–2.59) but not with Intermediate frailty, likely reflecting the possibility that severe impairment increases care requirements rather than care receipt being independently associated with frailty onset. Living alone was not independently associated with either frailty profile after adjustment for other covariates (Table 2).

E-value analyses indicated that key associations were robust to potential unmeasured confounding (Supplementary Table S14 in S1 File). For the association between poor self-rated health and severe frailty (OR 47.89), the E-value was 95.3, meaning an unmeasured confounder would need to be associated with both poor health and severe frailty by a risk ratio of at least 95-fold each to fully account for the observed association. For very poor self-rated health (OR 95.78), the E-value exceeded 191. Similarly, female sex (E-value 4.0 for severe frailty) and physical inactivity (E-value 3.6) showed E-values substantially exceeding their confidence interval bounds, indicating robustness. These findings suggest that the observed associations, particularly for self-rated health, are unlikely to be attributable to unmeasured confounding alone.

Hierarchical regression analyses indicated that health- and lifestyle-related variables accounted for the largest proportion of explained variance in frailty profile membership (51.9%), followed by demographic factors (39.8%). Collectively, these two domains explained more than 90% of the model’s total explanatory power. In contrast, socioeconomic characteristics contributed a comparatively modest incremental share of variance (3.8%) once health and lifestyle factors were included, suggesting that their associations with frailty profiles were substantially attenuated after adjustment for health-related variables rather than retaining independent explanatory power.

Environmental and household characteristics explained an additional 2.2% of the variance, while regional context contributed a further 2.2%. The fully adjusted model demonstrated substantial improvement over the null model (McFadden’s pseudo-R^2^ = 0.235), with consistent reductions in the Akaike Information Criterion across successive blocks, supporting the incremental contribution of each domain (Table 3).

**Table 3:** Incremental explanatory contribution of hierarchical predictor blocks to frailty profile membership from multinomial logistic regression.

Block	Predictor block *(variables added)*	McFadden pseudo-R²	∆R²	Share of final R² (%)	AIC	LRT *p*-value
1. Demographics	Age, sex, marital status	0.094	0.094	39.8	6,269,658	—
2. + Health & Lifestyle	Chronic disease, self-rated health, physical activity frequency, Living alone	0.216	0.122	51.9	6,389,348	<0.001
3. + Socioeconomic	Education, social security	0.225	0.009	3.8	6,389,859	<0.001
4. + Environmental/Household	Elevator access, internet access, home care need, household size	0.230	0.005	2.2	6,389,865	<0.001
5. + Regional context	NUTS-1 region *(12 categories)*	0.235	0.005	2.2	6,389,472	<0.001

Survey-weighted multinomial logistic regression with Robust profile as reference category. Variables entered in theoretically ordered blocks; each model includes all variables from preceding blocks. ΔR^2^: incremental change from preceding model. Share (%): proportion of final model R^2^ attributable to each block. AIC: Akaike Information Criterion. LRT: likelihood ratio test comparing successive nested models. Reference categories: male (sex), divorced/separated (marital status), no formal education (education), Istanbul (NUTS-1 region), very good (self-rated health), 1–3 times monthly (physical activity), no (all binary variables).

## Discussion

This nationally representative analysis indicates that 48.6% of adults aged 50 years and older in Türkiye exhibit some degree of frailty, a prevalence that is higher than estimates reported in many high-income settings and in previous Turkish studies. Siriwardhana and colleagues [6] reported a pooled frailty prevalence of 17.4% across low- and middle-income countries, while Akin and colleagues [9] identified a prevalence of 27.8% among community-dwelling older adults in Türkiye using modified Fried criteria (2001). The higher prevalence observed in the present study likely reflects differences in frailty operationalisation, as well as the limited availability of validated, multidimensional frailty screening instruments tailored to middle-income countries [7].

Using latent profile analysis, we identified three distinct multisystem frailty phenotypes—Robust (51.4%), Intermediate frailty (33.7%), and Severe multisystem frailty (14.8%)—that demonstrate substantial heterogeneity in functional impairment and risk factor profiles. This pattern is consistent with findings from Liu and colleagues [21], who reported clinically meaningful frailty subtypes using latent class approaches in Taiwan. Such heterogeneity reinforces the limitations of binary frailty classifications and supports a shift toward person-centred, data-driven assessment frameworks [2,5].

Importantly, the frailty profiles identified in this study were defined by concurrent impairments across motor, sensory, cognitive, and functional domains, rather than by the exclusively physical criteria of the Fried phenotype [4]. By integrating multiple motor, sensory, cognitive, and functional dimensions, this approach captures a broader, disability-adjacent construct of frailty that reflects cumulative vulnerability across interconnected systems. This multidomain perspective may help explain the higher prevalence estimates observed and aligns frailty more closely with cumulative multisystem decline rather than isolated physical weakness.

The comparison of the LPA-derived profiles with conventional frailty classifications provided direct evidence regarding the added value of the multisystem approach. When a modified Fried phenotype proxy—limited to three physical criteria—was applied to the same population, overall frailty prevalence was broadly comparable (49.2% vs 48.6%). However, cross-classification revealed that 4,441 individuals (16.5% of the sample) were identified as exhibiting multisystem frailty by LPA but classified as robust by the modified Fried proxy. These discordant cases exhibited sensory and cognitive composite scores of 15.0% and 28.6%, respectively, despite motor impairment below the Fried threshold. This finding directly demonstrates that physical phenotype-based criteria systematically overlook individuals whose vulnerability manifests primarily in sensory and cognitive domains—a subpopulation that may represent an early or alternative trajectory of frailty progression. In contrast, the Frailty Index, constructed using a deficit accumulation approach [18], classified 87.9% of the study population as having some degree of frailty. Although the FI incorporates non-physical domains, its reliance on cumulative deficit counts without distinguishing qualitatively distinct impairment profiles limits its capacity to identify clinically meaningful subgroups. These results are consistent with a growing body of evidence demonstrating substantial discordance in individual-level frailty classification across instruments. Xue and colleagues [22] reported that only 12% of older adults classified as frail by either the physical frailty phenotype or the Frailty Index were identified by both instruments, with age and disease burden as the strongest predictors of discordant classification. Similarly, Oviedo-Briones and colleagues [23] found only fair interscale agreement among eight commonly used frailty tools across European clinical settings, concluding that most instruments assess fundamentally different frailty constructs. Van der Elst and colleagues [24] further demonstrated that the characteristics of individuals classified as frail differ substantially depending on whether a unidimensional or multidimensional instrument is used (kappa = 0.35–0.45), with multidimensional approaches capturing social and psychological vulnerability that physical phenotype-based tools overlook. It should also be noted that part of the observed discordance across instruments may reflect measurement-level artifacts, such as differential item functioning or non-invariant factor structures across demographic subgroups, which can inflate apparent between-group heterogeneity beyond genuine population differences [25]. The present findings extend this evidence by showing that, in a nationally representative middle-income country sample, a data-driven multisystem approach identifies a specific subpopulation with sensory-cognitive vulnerability that is systematically missed by physical criteria, thereby providing empirical support for multidimensional, person-centred frailty assessment frameworks [4,18,26]. The inability to construct the full five-item Fried phenotype within this survey—owing to the absence of weight loss and exhaustion measure—itself underscores the practical constraints that secondary data analyses in middle-income settings face when applying instruments originally developed in well-resourced clinical cohorts, and further supports the rationale for data-driven, multisystem alternatives.

Hierarchical regression analyses indicated that demographic and health–lifestyle factors together accounted for more than 90% of the explained variance in frailty profile membership, with health and lifestyle characteristics alone contributing 51.9% after adjustment for demographic factors. In contrast, socioeconomic position explained only a modest additional proportion of variance (3.8%) once health-related variables were included. This pattern is consistent with prior research showing that health behaviours account for a substantial proportion of socioeconomic gradients in health outcomes, explaining approximately 40% of these differences, while lifestyle factors have also been shown to account for a measurable portion of socioeconomic disparities [27,28].

The dominance of health and lifestyle variables in explaining frailty variation has direct implications for screening and risk stratification. A substantial proportion of the information captured by socioeconomic indicators appears to be reflected in health status measures, particularly self-rated health. As an integrative assessment, self-rated health may summarise multisystem burden by capturing symptoms, functional limitations, and subclinical disease severity that are not fully represented by socioeconomic indicators alone. The exceptionally large odds ratios observed for poor and very poor self-rated health (OR range 42.94–87.56) should be interpreted in light of both the five-level ordinal scale, which compares extreme categories against the most favourable reference, and the inherent conceptual overlap between subjective health appraisal and the multisystem frailty construct itself. Self-rated health likely captures subclinical impairments and symptom burden that are also reflected in the frailty indicators, which may inflate the observed associations beyond what would be expected from a truly exogenous predictor. Taken together, these findings suggest that the association between socioeconomic position and frailty is substantially attenuated after accounting for health-related variables, indicating limited independent explanatory contribution of socioeconomic factors beyond health status.

Women exhibited substantially higher odds of frailty compared with men, a pattern consistent with previous evidence from Türkiye and other countries [9,29] and with the widely documented frailty–mortality paradox, whereby women experience higher frailty prevalence yet lower mortality risk. Evidence indicates the presence of sex-specific patterns in frailty progression, whereby weakness more frequently characterizes frailty onset among men, whereas exhaustion appears to play a more prominent role among women [30]. Similarly, Xue and colleagues [29] identified weakness as a primary entry point into the frailty cycle, whereas Romero-Ortuno and colleagues [31] demonstrated considerable cross-national variation in sex differences in frailty-free life expectancy. Together, these findings support the interpretation that sex disparities in frailty reflect not only differential exposure to risk factors but also distinct biological, behavioural, and social ageing processes.

Educational attainment showed a clear protective association, with individuals holding a university degree demonstrating substantially lower odds of frailty compared with those without formal education. This association is consistent with prior evidence indicating that education is linked to frailty risk partly through its relationship with health literacy and self-management capacities [32]. However, the relatively small incremental contribution of education to explained variance (3.8%) after accounting for health and lifestyle factors suggests that the association between socioeconomic position and frailty is largely captured by health-related variables rather than representing an independent domain of risk.

Living alone was conceptualised within the health–lifestyle domain, reflecting its role as a social health risk rather than a purely structural household characteristic. Its contribution to the health–lifestyle block is consistent with evidence indicating that social isolation affects health primarily through behavioural and psychosocial mechanisms. This finding contrasts with results from JAGES data showing that living arrangements may exert independent effects on health outcomes, suggesting that the vulnerability associated with living alone may be context specific [33]. This interpretation is further supported by evidence indicating that Mediterranean family structures may buffer the adverse consequences of solitary living through informal care networks and intergenerational support, potentially attenuating the association between household structure and frailty risk in Türkiye [34].

These findings have several implications for frailty prevention and management in Türkiye and comparable middle-income settings. Frailty is increasingly recognised as a potentially reversible condition when identified at earlier stages [2], which makes the choice of screening approach consequential. The substantial explanatory contribution of self-rated health observed in this study suggests that a single-item subjective health assessment, when considered alongside age and sex, may serve as a pragmatic and low-cost first-line screening tool in primary care. Importantly, however, the identification of sensory-cognitive vulnerability as a distinguishing feature of the intermediate frailty profile indicates that screening strategies focused exclusively on physical function—as emphasised in traditional Fried-based approaches—risk overlooking a sizeable at-risk subpopulation. Effective early detection therefore requires multidomain assessment that extends beyond motor performance to include routine vision and hearing evaluation, cognitive monitoring, and enquiry into subjective memory complaints.

These screening considerations have direct implications for intervention design. Evidence increasingly supports the effectiveness of multicomponent programmes that combine physical exercise with cognitive training, nutritional counselling, and social engagement in slowing or reversing frailty progression among community-dwelling older adults. The strong protective association observed for daily physical activity in this study (OR 0.49 for severe frailty) reinforces the potential of community-based exercise initiatives as a scalable, low-cost preventive strategy. For Türkiye specifically, embedding multidomain frailty screening within the existing primary care infrastructure—including family health centres and home-based care programmes—could facilitate early identification and timely, targeted intervention. The pronounced sex and age gradients in frailty prevalence further suggest that prevention efforts should prioritise women and adults approaching the 65-year threshold, among whom the transition from intermediate to severe frailty appears most concentrated. More broadly, the combination of self-reported, questionnaire-based indicators with data-driven profiling methods such as LPA offers a practical pathway for population-level frailty surveillance in settings where performance-based clinical measures are not routinely available, and may serve as a model for other countries facing accelerated population ageing alongside constrained health system resources.

The use of nationally representative data from more than 26,000 adults aged 50 years and older enhances the generalisability of the findings to the broader Turkish population. The availability of detailed sociodemographic, socioeconomic, health-related, and environmental information enabled comprehensive covariate adjustment. The hierarchical modelling strategy allowed for quantification of the relative and unique contributions of different predictor domains to frailty profile membership, providing a transparent variance decomposition framework with direct policy relevance. The multisystem approach adopted in this study captures a broader spectrum of vulnerability than traditional phenotypic definitions focused solely on physical components, and may offer added value for understanding complex frailty processes in ageing populations [35].

Several limitations should be acknowledged. First, the cross-sectional design precludes causal inference and limits conclusions regarding temporal sequencing between covariates and frailty profile membership. Second, reliance on self-reported measures may introduce recall or reporting bias; however, prior work supports the reasonable validity of self-reported frailty-related measures in older populations [36,37]. Third, sensitivity analyses using E-values indicated that the observed associations were robust to potential unmeasured confounding, particularly for self-rated health, for which an unmeasured confounder would need to exhibit implausibly strong associations with both exposure and outcome to fully account for the findings.

Fourth, the near-perfect entropy value (1.00) observed in the three-profile solution warrants careful interpretation. Inspection of within-class indicator distributions revealed that walking difficulty exhibited zero within-class variance in two of the three profiles, indicating that this variable effectively determined profile membership under the equal-variance, zero-covariance parameterisation. This pattern was replicated among women and adults aged 65 years and older but not among adults aged 50–64 years (entropy = 0.938), suggesting that the degree of class separation is partly age-dependent. The deterministic classification may reflect the strong discriminating power of motor impairment in this population, but it also implies that the identified profiles are primarily driven by walking difficulty rather than emerging from a balanced contribution of all eight indicators. This represents a limitation of the equal-variance constraint when applied to indicators with heterogeneous measurement scales and distributional properties.

Fifth, the latent profile analysis treated ordinal and binary indicators as continuous, assuming within-class normality. Although this approach is common in applied research, it may produce biased parameter estimates when indicators have limited response categories. Sixth, participants with missing data on the subjective memory complaints item (n = 2,880; 9.7%) were excluded rather than imputed, as combining multiple imputation with latent profile analysis requires specialised procedures that are not well established in the survey-weighted context. Although comparisons between included and excluded participants revealed no substantial systematic differences (Supplementary Material S1 in S1 File), the possibility of non-ignorable missingness cannot be excluded.

Future studies could address the measurement and classification limitations noted above by employing latent class analysis with categorical variable specification, by using continuous indicators with greater distributional overlap across classes, and by applying recently developed methods for integrating multiple imputation with mixture models.

## Supporting information

S1 File Supplementary material. Comprehensive supplementary information including missing value analysis and sample characteristics (**Table S1**), detailed description of study measures (**Table S2**), sample characteristics by age group (**Table S3**), pre-analysis diagnostics (**Fig S1**), latent profile analysis model fit indices (**Table S4**), profile characteristics and demographic distribution (**Tables S5–S6**), sensitivity analyses (**Tables S7, S9–S10**), measurement invariance assessment (**Section 8**), hierarchical regression results (**Tables S11–S12**), comparison with conventional frailty classifications (**Table S13**), E-value sensitivity analysis (**Table S14**), and statistical software details (**Table S15**).
(DOCX)
