# Supplementary material for: Multisystem frailty phenotypes and associated factors among older adults in Türkiye: A nationally representative study
Source: PLoS One. 2026 Jun 26;21(6):e0352767. doi: 10.1371/journal.pone.0352767 (PMC13308790; doi:10.1371/journal.pone.0352767)
Supplement: S1 File — Comprehensive supplementary information including missing value analysis and sample characteristics (Table S1), detailed description of study measures (Table S2), sample characteristics by age group (Table S3), pre-analysis diagnostics (Fig S1), latent profile analysis model fit indices (Table S4), profile characteristics and demographic distribution (Tables S5–S6), sensitivity analyses (Tables S7, S9–S10), measurement invariance assessment (Section 8), hierarchical regression results (Tables S11–S12), comparison with conventional frailty classifications (Table S13), E-value sensitivity analysis (Table S14), and statistical software details (Table S15). (DOCX) [file pone.0352767.s001.docx]

**Supplementary Information for:**

***Multisystem frailty phenotypes and associated factors among older adults in Türkiye: a nationally representative study***

**Salim Yılmaz, Yusuf Çelik, Elif Cansu Kara**

**Corresponding author**: Salim Yılmaz,

**Email:** [salim.yilmaz@acibadem.edu.tr](mailto:salim.yilmaz@acibadem.edu.tr)

**Supplementary Materials**

**Multisystem frailty phenotypes and associated factors among older adults in Türkiye: a nationally representative study**

This supplementary material offers comprehensive information on data quality assessment and sample selection procedures for the study investigating multisystem frailty phenotypes among middle-aged and older adults in Türkiye. The analysis utilised data from the 2023 Turkish Statistical Institute (TURKSTAT) Elderly Statistics Survey, a nationally representative cross-sectional survey. The original dataset comprised 29,785 individuals aged ≥50 years. This section documents missing data patterns and compares the characteristics of included versus excluded participants to assess potential selection bias.

| **Abbreviations** |  |
| --- | --- |
| ADLs: | Activities of Daily Living |
| AIC: | Akaike Information Criterion |
| BIC: | Bayesian Information Criterion |
| BLRT: | Bootstrap Likelihood Ratio Test |
| CI: | Confidence Interval |
| DEFF: | Design Effect |
| FI: | Frailty Index |
| FIML: | Full-Information Maximum Likelihood |
| GDS: | Geriatric Depression Scale |
| ICC: | Intraclass Correlation Coefficient |
| IQR: | Interquartile Range |
| LPA: | Latent Profile Analysis |
| LRT: | Likelihood Ratio Test |
| MAR: | Missing at Random |
| MCAR: | Missing Completely at Random |
| MNAR: | Missing Not at Random |
| NUTS-1: | Nomenclature of Territorial Units for Statistics, Level 1 |
| OR: | Odds Ratio |
| SD: | Standard Deviation |
| SE: | Standard Error |
| saBIC: | Sample Size-Adjusted Bayesian Information Criterion |
| TURKSTAT: | Turkish Statistical Institute |
| WG-SS: | Washington Group Short Set on Functioning |

**Contents and List of Supplementary Tables and Figures**

[Section 1: Missing Value Analysis and Sample Characteristics 1](#_Toc228544544)

[Table S1. Missing data patterns and sample characteristics by analytic inclusion status 1](#_Toc228544545)

[Section 2: Detailed Description of Study Measures 2](#_Toc228544546)

[**2.1 Multisystem frailty indicators** 2](#_Toc228544547)

[**2.2 Covariates** 2](#_Toc228544548)

[Table S2. Covariate definitions and coding 3](#_Toc228544549)

[**2.3 Survey weights** 3](#_Toc228544550)

[Section 3: Sample Characteristics by Age Group 4](#_Toc228544551)

[Table S3. Sample characteristics by age group (survey-weighted) 4](#_Toc228544552)

[Section 4: Pre-analysis Diagnostics 5](#_Toc228544553)

[Fig S1. Polychoric correlation matrix among multisystem frailty indicators 5](#_Toc228544554)

[Section 5: Model Fit Indices for Latent Profile Analysis Model Selection 6](#_Toc228544555)

[Table S4. Fit indices for latent profile models 6](#_Toc228544556)

[Section 6: Profile Characteristics and Distribution Across Demographic Subgroups 7](#_Toc228544557)

[Table S5. Characteristics of the three multisystem frailty profiles 7](#_Toc228544558)

[Table S6. Survey-weighted prevalence of multisystem frailty profiles by demographic subgroups 7](#_Toc228544559)

[Section 7: Weighted Multinomial Regression without Survey Design Correction 9](#_Toc228544560)

[Table S7. Weighted multinomial logistic regression for multisystem frailty profiles (sensitivity analysis without survey design correction) 10](#_Toc228544561)

[Section 8: Measurement Invariance Assessment 11](#_Toc228544562)

[Section 9: Subgroup-stratified Multinomial Regression Analyses 12](#_Toc228544563)

[Table S9. Sex-stratified survey-weighted multinomial logistic regression 12](#_Toc228544564)

[Table S10. Age-stratified survey-weighted multinomial logistic regression 13](#_Toc228544565)

[Section 10: Hierarchical Multinomial Logistic Regression with Nested Predictor Blocks 14](#_Toc228544566)

[Table S11. Assessment of clustering structure for consideration of multilevel modelling 14](#_Toc228544567)

[Table S12. Full model results from hierarchical multinomial logistic regression (Model 5) for multisystem frailty profiles 17](#_Toc228544568)

[Section 11: Comparison with Conventional Frailty Classifications 18](#_Toc228544569)

[Table S13. Comparison of frailty classification approaches: survey-weighted prevalence and cross-classification 18](#_Toc228544570)

[Section 12: Sensitivity Analysis for Unmeasured Confounding Using E-values 20](#_Toc228544571)

[Table S14. E-values for sensitivity to unmeasured confounding in associations with severe multisystem frailty 20](#_Toc228544572)

[Section 13: Statistical Software and Reproducibility 21](#_Toc228544573)

[Table S15. R packages used for statistical analysis 21](#_Toc228544574)

[References for Supplementary Material 22](#_Toc228544575)

Section 1: Missing Value Analysis and Sample Characteristics

Latent profile analysis (LPA), a person-centred clustering approach, was employed to identify multisystem frailty phenotypes.(Oberski 2016) LPA was conducted using eight indicator variables capturing four functional domains: motor function (difficulty walking, difficulty grasping, and falls), sensory function (difficulty seeing and hearing), cognitive function (difficulty learning and subjective memory complaints), and functional dependence (ADL dependency).

Table S1 presents the missing data patterns for these indicators and compares demographic and health characteristics between participants included in and excluded from the analytic sample.

**Table S1. Missing data patterns and sample** **characteristics by analytic inclusion status**

| **Domain** | **Variable** | **n (Missing)** | **% (Missing)** |
| --- | --- | --- | --- |
| **Motor** | Difficulty walking | 0 | 0.00 |
|  | Difficulty grasping | 0 | 0.00 |
|  | Falls (past 12 months) | 0 | 0.00 |
| **Sensory** | Difficulty seeing | 0 | 0.00 |
|  | Difficulty hearing | 0 | 0.00 |
| **Cognitive** | Difficulty learning | 0 | 0.00 |
|  | Subjective memory complaints | 2880 | 9.67 |
| **Functional** | ADL dependency | 0 | 0.00 |
| **Characteristic** | **Included (n = 26,905)** | **Excluded (n = 2880)** | ***p*-value** |
| **Age, mean (SD)** | 62.9 (9.4) | 65.8 (12.1) | <0.001 |
| **Age group** |  |  |  |
| 50–64 years | 61.5% | 54.5% | <0.001 |
| ≥ 65 years | 38.5% | 45.5% |  |
| **Sex** |  |  |  |
| Female | 53.0% | 51.5% | 0.120 |
| Male | 47.0% | 48.5% |  |
| **Living alone** | 12.6% | 6.2% | <0.001 |
| **Chronic disease** | 64.7% | 65.7% | 0.296 |
| **ADL score, mean (SD)** | 5.77 (0.82) | 5.07 (1.52) | <0.001 |

ADLs, activities of daily living (range, 0–6; higher scores indicate greater independence). p-values are derived from χ² tests for categorical variables. For continuous variables, Welch’s t test is used owing to heterogeneity of variances (Levene’s test: F = 404.38, p < 0.001). Considering the large sample sizes, the central limit theorem supports the robustness of parametric tests despite non-normal distributions (Shapiro–Wilk p < 0.001). Results are confirmed using Mann–Whitney U tests, yielding identical conclusions (p < 0.001 for age and ADL score).

Missing data were present exclusively in the subjective memory complaints variable, which is derived from the Geriatric Depression Scale (GDS). Seven of the eight LPA indicators had complete data (0% missing), whereas memory complaints had 9.67% missingness (n = 2880). Comparison of included versus excluded participants revealed systematic differences. Participants with missing data were significantly older (65.8 vs 62.9 years, p < 0.001), had lower ADL scores (5.07 vs 5.77, p < 0.001), and were less likely to live alone (6.2% vs 12.6%, p < 0.001). No significant differences were observed for sex (p = 0.120) or chronic disease prevalence (p = 0.296). These results indicate that missingness is unlikely to be completely at random (MCAR) and may reflect underlying frailty, including cognitive limitations or dependence on proxy respondents who were unable to answer GDS items. LPA models were estimated using full-information maximum likelihood, which provides unbiased estimates under missing-at-random (MAR) assumptions. However, when missingness is driven by unmeasured cognitive impairment (e.g., missing not at random [MNAR]), the prevalence of the most impaired multisystem frailty profiles may be underestimated, suggesting conservative classification (Table S1).

Section 2: Detailed Description of Study Measures

2.1 Multisystem frailty indicators

***Motor function domain***

Difficulty walking and grasping objects were assessed using the Washington Group Short Set on Functioning (WG-SS), a standardised UN-endorsed instrument for internationally comparable disability measurement.(Madans et al. 2011) Response categories ranged from 0 (“no difficulty”) to 3 (“cannot do at all”). Falls were assessed using a binary item asking whether the respondent had fallen in the past 12 months (0 = no, 1 = yes).

***Sensory function domain***

Difficulty seeing (with glasses, if used) and hearing (with hearing aids, if used) were obtained from the WG-SS using the same four-level difficulty scale (0–3).

***Cognitive function domain***

Using the WG-SS item on memory/concentration difficulties, difficulty learning new tasks was measured.
Subjective memory complaints were drawn from Item 14 of the GDS-30:(Yesavage et al. 1982) “*Do you feel you have more problems with memory than most people?*” (0 = no, 1 = yes). The Turkish GDS has demonstrated adequate psychometric properties.(Ertan and Eker 2000)

***Functional dependence domain***

Activities of Daily Living (ADLs) were assessed using the Katz Index,(Katz et al. 1963) encompassing bathing, dressing, toileting, transferring, continence, and feeding. Each activity was scored 1 (independent) or 0 (dependent). Total scores ranged from 0 to 6; ADL dependency was defined as any limitation (score < 6) and coded as 1 (dependent).

2.2 Covariates

Table S2 provides a summary of covariate definitions and coding.

**Table S2. Covariate definitions and coding**

| **Variable** | **Definition** | **Coding** |
| --- | --- | --- |
| ***Demographics*** |  |  |
| Age | Chronological age at survey completion, calculated from date of birth | Continuous (years);  categorical: 50–64, ≥ 65 |
| Sex | Biological sex as recorded in the population registry | 0 = Male, 1 = Female |
| Marital status | Legal marital status during interview | 1 = Married, 2 = Widowed,  3 = Divorced, 4 = Never married |
| Education | Highest level of formal education completed | 1 = No formal education,  2 = Primary school,  3 = Secondary/High school,  4 = Higher education |
| Region | NUTS Level-1 statistical region of residence | TR1–TR12 (12 regions) |
| ***Socioeconomics*** |  |  |
| Individual income | Self-reported monthly personal income from all sources (wages, pensions, and transfers) | Quintiles (1–5) |
| Social security | Coverage by any social security scheme | 0 = No coverage, 1 = Has coverage |
| Living alone | Respondent solely occupies the household | 0 = No, 1 = Yes |
| Household size | Total number of individuals residing in the household | Continuous |
| ***Health-related*** |  |  |
| Chronic disease | Presence of any physician-diagnosed chronic condition (e.g., hypertension, diabetes, and cardiovascular disease) | 0 = No, 1 = Yes |
| Self-rated health | Subjective assessment of overall health status | 1 = Very good, 2 = Good,  3 = Fair, 4 = Poor, 5 = Very poor |
| Smoking | Current tobacco use status | 0 = Never/Former,  1 = Current (daily or occasional) |
| Physical activity | Frequency of leisure-time physical activity | 1 = Never,  2 = Occasional (1–3 times/month),  3 = Regular (weekly or daily) |
| ***Environmental*** |  |  |
| Elevator access | Presence of a functioning elevator in the residential building | 0 = No, 1 = Yes |
| Internet access | Household has internet connection (fixed or mobile) | 0 = No, 1 = Yes |
| Care need | Self-reported need for assistance with home-based personal care or daily activities | 0 = No, 1 = Yes |

2.3 Survey weights

Individual-level sampling weights (*FAKTOR_FERT*) were provided by Turkish Statistical Institute (TURKSTAT) to adjust for the stratified two-stage cluster design. Weights account for:

- differential selection probabilities,
- nonresponse adjustments, and
- poststratification calibration to national population totals.

The original sample (N = 29**,**785) represents approximately 22.4 million adults aged ≥50 years in Türkiye. After excluding participants with missing data on LPA indicator variables (n = 2880; 9.7%), the analytic sample (n = 26**,**905) represents approximately 20.2 million adults.

Section 3: Sample Characteristics by Age Group

Compared with adults aged 50–64 (median, 57; interquartile range [IQR], 53–60) years, those aged ≥65 (median, 71; IQR, 68–77) years were more likely to be females (55.1% vs 51.2%), widowed (32.0% vs 6.6%), living alone (21.5% vs 8.0%), and have no formal education (30.6% vs 11.4%). Older adults had a smaller median household size (2 vs 3 persons). Age groups demonstrated comparable social security coverage (87.0% vs 87.9%). Older adults (78.3% vs 55.2%) and those with poor or very poor self-rated health (26.1% vs 13.1%) demonstrated a substantially higher chronic disease prevalence. Older adults were less likely to use tobacco daily (11.8% vs 27.9%), engage in daily physical activity (19.1% vs 23.8%), have elevator access (23.1% vs 27.9%), or have internet access (40.3% vs 66.9%). The older group showed markedly higher home care needs (14.6% vs 5.6%, all p < 0.001) (Table S3).

**Table S3. Sample characteristics by age group (survey-weighted)**

| **Characteristic** | **Overall**  *(N = 20,174,792)* | **50–64 years**  *(n = 12,570,708)* | **≥ 65 years**  *(n = 7,604,084)* | **p-value** |
| --- | --- | --- | --- | --- |
| **Age, years, mean (SD)** | 62.7 (9.4) | 56.6 (4.3) | 72.8 (6.4) | <0.001 |
| **Sex, n (%)** |  |  |  |  |
| Males | 9,549,321 (47.3) | 6,136,909 (48.8) | 3,412,412 (44.9) | <0.001 |
| Females | 10,625,471 (52.7) | 6,433,799 (51.2) | 4,191,672 (55.1) |  |
| **Marital status, n (%)** |  |  |  |  |
| Married | 15,410,136 (76.4) | 10,602,176 (84.4) | 4,807,960 (63.2) | <0.001 |
| Widowed | 3,261,157 (16.2) | 825,885 (6.6) | 2,435,272 (32.0) |  |
| Divorced | 1,021,687 (5.1) | 762,255 (6.1) | 259,432 (3.4) |  |
| Never married | 476,009 (2.4) | 375,934 (3.0) | 100,075 (1.3) |  |
| **Education, n (%)** |  |  |  |  |
| No formal education | 3,760,103 (18.6) | 1,435,894 (11.4) | 2,324,209 (30.6) | <0.001 |
| Primary school | 10,026,797 (49.7) | 6,468,389 (51.5) | 3,558,408 (46.8) |  |
| Secondary school | 1,637,922 (8.1) | 1,202,174 (9.6) | 435,748 (5.7) |  |
| High school | 2,501,646 (12.4) | 1,876,232 (14.9) | 625,414 (8.2) |  |
| Higher education | 2,248,324 (11.1) | 1,588,019 (12.6) | 660,305 (8.7) |  |
| **Living alone, n (%)** |  |  |  |  |
| Yes | 2,640,235 (13.1) | 1,007,439 (8.0) | 1,632,796 (21.5) | <0.001 |
| **Household size, mean (SD)** | 3.1 (1.8) | 3.3 (1.7) | 2.7 (1.8) | <0.001 |
| **Social security coverage, n (%)** |  |  |  |  |
| Yes | 17,625,106 (87.4) | 10,938,053 (87.0) | 6,687,053 (87.9) | 0.038 |
| **Chronic disease, n (%)** |  |  |  |  |
| Yes | 12,892,062 (63.9) | 6,936,028 (55.2) | 5,956,034 (78.3) | <0.001 |
| **Self-rated health, n (%)** |  |  |  |  |
| Very good | 511,434 (2.5) | 414,676 (3.3) | 96,759 (1.3) | <0.001 |
| Good | 6,609,708 (32.8) | 4,919,632 (39.1) | 1,690,076 (22.2) |  |
| Fair | 9,425,679 (46.7) | 5,590,286 (44.5) | 3,835,394 (50.4) |  |
| Poor | 3,267,051 (16.2) | 1,497,072 (11.9) | 1,769,979 (23.3) |  |
| Very poor | 360,919 (1.8) | 149,043 (1.2) | 211,876 (2.8) |  |
| **Tobacco use, n (%)** |  |  |  |  |
| Daily | 4,406,099 (21.8) | 3,511,923 (27.9) | 894,176 (11.8) | <0.001 |
| Occasional | 600,712 (3.0) | 448,208 (3.6) | 152,504 (2.0) |  |
| Former | 3,481,390 (17.3) | 1,863,574 (14.8) | 1,617,816 (21.3) |  |
| Never | 11,686,591 (57.9) | 6,747,003 (53.7) | 4,939,588 (65.0) |  |
| **Physical activity, n (%)** |  |  |  |  |
| Daily or almost daily | 4,451,972 (22.1) | 2,996,917 (23.8) | 1,455,055 (19.1) | <0.001 |
| At least weekly | 2,525,503 (12.5) | 1,708,132 (13.6) | 817,370 (10.7) |  |
| 1–3 times monthly | 857,609 (4.3) | 588,683 (4.7) | 268,925 (3.5) |  |
| Rarely | 3,800,466 (18.8) | 2,484,025 (19.8) | 1,316,441 (17.3) |  |
| Never | 8,539,243 (42.3) | 4,792,951 (38.1) | 3,746,292 (49.3) |  |
| **Elevator access, n (%)** |  |  |  |  |
| Yes | 5,269,578 (26.1) | 3,509,947 (27.9) | 1,759,631 (23.1) | <0.001 |
| **Internet access, n (%)** |  |  |  |  |
| Yes | 11,478,145 (56.9) | 8,412,745 (66.9) | 3,065,401 (40.3) | <0.001 |
| **Home care needs, n (%)** |  |  |  |  |
| Yes | 1,811,490 (9.0) | 704,820 (5.6) | 1,106,670 (14.6) | <0.001 |

Data are weighted n (%) or mean (standard deviation [SD]). p-values are from design-based Rao–Scott χ² and Wald tests for categorical and continuous variables, respectively.

Section 4: Pre-analysis Diagnostics

Before LPA, polychoric correlations among indicator variables were investigated to assess multicollinearity and confirm that indicators captured sufficiently distinct constructs for profile identification. Figure S1 depicted polychoric correlations among the eight multisystem frailty indicators.


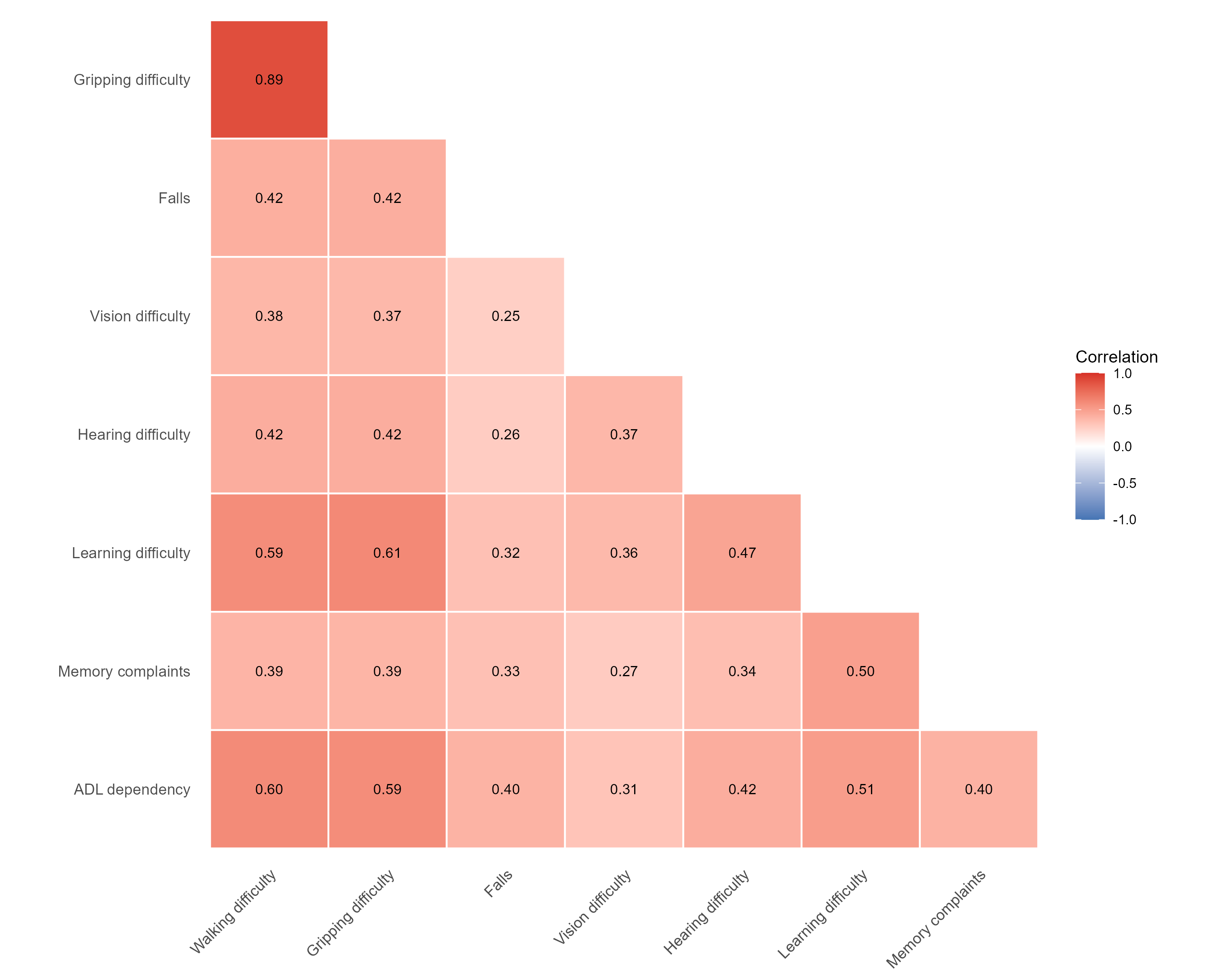


Fig S1. Polychoric correlation matrix among multisystem frailty indicators

Walking difficulty and gripping difficulty demonstrated the highest correlation (r = 0.89), consistent with their shared motor domain. Other moderate correlations were noted between motor indicators and ADL dependency (r = 0.59–0.60) and between learning difficulty and other indicators (r = 0.32–0.61). Sensory indicators (vision and hearing) revealed weaker correlations with other domains (r = 0.25–0.47). Memory complaints were moderately associated with learning difficulty (r = 0.50) but weakly correlated with motor and sensory indicators (r = 0.27–0.39). The correlation between walking and gripping difficulty exceeded the conventional threshold of 0.85; however, both indicators were retained because they capture distinct aspects of motor function (mobility vs dexterity), and removing either would reduce content validity. No other indicator pair exceeded r = 0.70, reinforcing the inclusion of all eight indicators in the LPA (Fig S1).

Section 5: Model Fit Indices for Latent Profile Analysis Model Selection

Latent profile models specifying 2–7 classes were estimated under two variance parameterizations: (1) equal within-class variances with zero covariances and (2) varying within-class variances with zero covariances. Models with varying variances failed to converge for all class solutions (K = 2–7), yielding inadmissible solutions. Within the equal-variance family, models with 4–7 classes also failed to converge. Therefore, model comparison was restricted to admissible two- and three-class solutions from the equal-variance specification.

**Table S4. Fit indices for latent profile models**

| **Classes** | **LogLik** | **AIC** | **BIC** | **ssaBIC** | **Entropy** | **BLRT p** | **AvePP min** | **AvePP max** | **n min (%)** | **n max (%)** |
| --- | --- | --- | --- | --- | --- | --- | --- | --- | --- | --- |
| 2 | **−152,237** | **304,525** | **304,730** | **304,650** | **0.851** | **0.01** | **0.926** | **0.968** | **29.7** | **70.3** |
| 3 | **−120,527** | **241,123** | **241,402** | **241,294** | **1.000** | **0.01** | **1.000** | **1.000** | **15.2** | **51.4** |
| **4–7** | — | — | — | — | — | — | — | — | — | — |

Models with 4–7 classes under the equal-variance specification and all models under the varying-variance specification failed to converge. AIC, Akaike Information Criterion; BIC, Bayesian Information Criterion; ssaBIC, sample-size adjusted BIC; BLRT, Bootstrap Likelihood Ratio Test; AvePP, average posterior probability

The three-class solution was selected on the basis of substantially lower information criteria (ΔAIC = 63**,**402; ΔBIC = 63**,**328), perfect classification accuracy (entropy = 1.00; all AvePP = 1.00), a significant BLRT (p = 0.01), an adequately sized smallest class (15.2%), and strong substantive interpretability. The identified profiles were labelled robust (51.4%), intermediate frailty (33.7%), and severe multisystem frailty (14.8%).

As a robustness check, LPA was repeated using standardised (z-score transformed) indicator variables. Results were consistent with the unstandardised analysis: the two- and three-class solutions successfully converged, whereas models with four to six classes unsuccessfully converged. The three-class solution again demonstrated superior fit (AIC = 503**,**667; BIC = 503**,**946; entropy = 1.00), and class membership assignments were identical to those obtained from the unstandardised analysis. These findings indicate that model selection is insensitive to variable scaling and supports the robustness of the three-class solution.

The three profiles, including robust (51.4%), intermediate frailty (33.7%), and severe multisystem frailty (14.8%), were defined on the basis of their indicator patterns (see Fig 2 in the main manuscript).

Section 6: Profile Characteristics and Distribution Across Demographic Subgroups

Design-based Wald tests of association were conducted using survey-weighted data to evaluate whether the distribution of multisystem frailty profiles significantly differed across demographic subgroups.

The three multisystem frailty profiles demonstrated distinct patterns across all assessed domains (Table S5).

**Table S5. Characteristics of the three multisystem frailty profiles**

| **Characteristic** | **Robust** | **Intermediate** | **Severe** |
| --- | --- | --- | --- |
| Sample size, n (unweighted %) | 13,556 (50.4) | 9270 (34.5) | 4079 (15.2) |
| Weighted prevalence, % (95% CI) | 51.4 (50.8–52.1) | 33.7 (33.1–34.3) | 14.8 (14.4–15.3) |
| **Motor domain** |  |  |  |
| Walking difficulty, mean (SD) | 0.00 (0.00) | 1.00 (0.00) | 2.06 (0.23) |
| Gripping difficulty, mean (SD) | 0.15 (0.41) | 0.98 (0.51) | 1.92 (0.61) |
| Falls in the past 12 months, % | 8.8 | 20.9 | 38.8 |
| **Sensory domain** |  |  |  |
| Vision difficulty, mean (SD) | 0.39 (0.54) | 0.63 (0.59) | 0.89 (0.74) |
| Hearing difficulty, mean (SD) | 0.14 (0.39) | 0.35 (0.56) | 0.58 (0.72) |
| **Cognitive domain** |  |  |  |
| Learning difficulty, mean (SD) | 0.16 (0.43) | 0.56 (0.66) | 0.98 (0.85) |
| Memory complaints, % | 25.3 | 46.6 | 60.7 |
| **Functional domain** |  |  |  |
| ADL dependency, % | 2.8 | 11.2 | 36.5 |
| **Prevalence by sex, %** |  |  |  |
| Males | 62.1 | 28.7 | 9.1 |
| Females | 41.8 | 38.2 | 20.0 |
| **Prevalence by age group, %** |  |  |  |
| 50–64 years | 62.1 | 29.0 | 8.9 |
| ≥ 65 years | 33.8 | 41.5 | 24.7 |

Mean scores for ordinal indicators range from 0 (no difficulty) to 3 (cannot perform at all). Percentages are unweighted for indicator distributions and weighted for prevalence estimates. ADLs, Activities of Daily Living; CI, confidence interval; SD, standard deviation

The robust profile, comprising approximately 50% of the sample (n = 13**,**556; weighted prevalence, 51.4%), was characterised by minimal impairment across all indicators, with near-zero walking difficulty (mean, 0.00), low gripping difficulty (mean, 0.15), and very low ADL dependency (2.8%). Compared with the robust profile, the intermediate profile (n = 9270; 33.7%) exhibited moderate deficits, with uniform walking difficulty (mean, 1.00), increased gripping impairment (mean, 0.98), and increased fall prevalence (20.9%). Memory complaints impacted approximately 50% of this group (46.6%), and ADL dependency was observed in 11.2% of the participants.

The severe profile (n = 4079; 14.8%) demonstrated significant impairment across all domains, including pronounced walking (mean, 2.06) and gripping difficulties (mean, 1.92), high fall prevalence (38.8%), and substantial ADL dependency (36.5%). The severe profile exhibited the most prevalent cognitive complaints, with 60.7% of the participants reporting memory difficulties. Sensory impairments showed a consistent gradient, with vision difficulty increasing from 0.39 (robust) to 0.89 (severe) and hearing difficulty from 0.14 to 0.58 across profiles.

These results reveal that the latent profiles capture meaningful gradients in multisystem frailty, with each successive profile reflecting progressively greater impairment across motor, sensory, cognitive, and functional domains (Table S5).

Design-based Wald tests of association were conducted using survey-weighted data to evaluate whether the distribution of multisystem frailty profiles varied across demographic subgroups (Table S6).

**Table S6. Survey-weighted prevalence of multisystem frailty profiles by demographic subgroups**

| **Subgroup** | **Robust % (95% CI)** | **Intermediate % (95% CI)** | **Severe % (95% CI)** |
| --- | --- | --- | --- |
| **Overall** | 51.4 (50.8–52.1) | 33.7 (33.1–34.3) | 14.8 (14.4–15.3) |
| **Sex** |  |  |  |
| Males *(ref)* | 62.1 (61.2–63.1) | 28.7 (27.9–29.6) | 9.1 (8.6–9.7) |
| Females | 41.8 (40.9–42.7) | 38.2 (37.3–39.1) | 20.0 (19.3–20.7) |
| **Age group** |  |  |  |
| 50–64 years *(ref)* | 62.1 (61.3–62.9) | 29.0 (28.3–29.8) | 8.9 (8.4–9.3) |
| ≥65 years | 33.8 (32.8–34.8) | 41.5 (40.5–42.5) | 24.7 (23.8–25.6) |
| **Sex × Age group** |  |  |  |
| Males 50–64 years *(ref)* | 71.6 (70.5–72.7) | 23.1 (22.1–24.1) | 5.3 (4.8–5.9) |
| Males ≥65 years | 45.1 (43.6–46.6) | 38.9 (37.4–40.4) | 16.0 (14.8–17.1) |
| Females 50–64 years | 53.1 (51.9–54.2) | 34.7 (33.6–35.8) | 12.2 (11.5–13.0) |
| Females ≥65 years | 24.5 (23.3–25.8) | 43.6 (42.2–45.0) | 31.9 (30.6–33.2) |

Data are survey-weighted percentages with 95% confidence intervals (CIs). Overall associations are assessed using design-based Wald tests (sex: F = 559.25, df = 2; age group: F = 1022.2, df = 2; sex × age group: F = 510.16, df = 6; all p < 0.001; denominator df = 26**,**904 for all tests). Pairwise comparison of severe frailty prevalence between females and males: Z = 23.79, p < 0.001.

Profile distribution significantly differed by sex (F = 559.25, df = 2, p < 0.001), age group (F = 1022.2, df = 2, p < 0.001), and the sex–age group interaction (F = 510.16, df = 6, p < 0.001). Females demonstrated a substantially higher severe frailty prevalence than males (20.0% vs 9.1%; absolute difference, 10.9 percentage points; Z = 23.79, p < 0.001), accompanied by a correspondingly lower robust profile prevalence (41.8% vs 62.1%). Adults aged ≥65 years exhibited markedly higher severe frailty prevalence than those aged 50–64 years (24.7% vs 8.9%), alongside a lower robust status prevalence (33.8% vs 62.1%). Intersectional analysis revealed that females aged ≥65 years had the highest severe frailty prevalence (31.9%), approximately six-fold higher than that observed among males aged 50–64 years (5.3%) (Table S6).

Section 7: Weighted Multinomial Regression without Survey Design Correction

We estimated a weighted multinomial logistic regression model using sampling weights (FAKTOR_FERT) but without applying survey design correction for standard errors (nnet::multinom) to evaluate the robustness of multisystem frailty profile membership predictors. In this specification, weights function as frequency weights rather than design weights; therefore, clustering and stratification effects are not incorporated. Results are provided for comparison with the main survey-weighted analysis presented in Table 2 of the main manuscript.

Findings were qualitatively consistent with the primary analysis. Females were associated with higher intermediate and severe multisystem frailty odds than males, and age revealed a clear graded effect, with increasing odds of both frailty profiles across advancing age. Education demonstrated a strong protective gradient, with individuals with higher educational attainment showing substantially lower intermediate and severe frailty odds than those with no formal education. Lack of social security coverage was consistently associated with elevated odds of both frailty profiles (Table S7).

Self-rated health exhibited the largest effect sizes. Compared with very good health, poor self-rated health was associated with markedly higher intermediate and severe frailty odds, whereas very poor health was associated with relatively lower intermediate frailty odds but dramatically higher severe frailty odds. This pattern mirrors the main analysis, suggesting that individuals with the worst perceived health status are disproportionately classified in the severe rather than the intermediate profile. Chronic disease presence and physical inactivity were associated with higher odds of severe frailty, whereas daily physical activity showed a protective association across frailty profiles. Home care needs were strongly associated with severe frailty but weakly inversely associated with intermediate frailty (Table S7).

The extremely narrow confidence intervals (CIs) and uniformly significant p-values observed in this sensitivity analysis reflect the use of model-based standard errors that do not account for the complex survey design. Accordingly, statistical inference should depend on the survey-weighted multinomial regression results presented in the main analysis (see main manuscript, Table 2), which offer appropriate variance estimation for nationally representative data.

**Table S7. Weighted multinomial logistic regression for multisystem frailty profiles (sensitivity analysis without survey design correction)**

| **Variable** | **Intermediate vs Robust** | | **Severe vs Robust** | | **p (overall)** |
| --- | --- | --- | --- | --- | --- |
|  | **OR (95% CI)** | **p-value** | **OR (95% CI)** | **p-value** |  |
| ***Demographics*** | | | | |  |
| **Age** (per year) | 1.05 (1.05–1.05) | <0.001 | 1.08 (1.08–1.08) | <0.001 | <0.001 |
| **Sex** (Females vs *Males*) | 1.59 (1.59–1.59) | <0.001 | 2.25 (2.24–2.26) | <0.001 | <0.001 |
| **Marital status** *(ref: Divorced)* | | | | |  |
| Widowed | 1.24 (1.23–1.24) | <0.001 | 1.32 (1.30–1.33) | <0.001 | <0.001 |
| Married | 1.17 (1.17–1.18) | <0.001 | 1.07 (1.06–1.08) | <0.001 |  |
| Never married | 0.77 (0.76–0.78) | <0.001 | 0.85 (0.84–0.86) | <0.001 |  |
| ***Socioeconomics factors*** |  |  |  |  |  |
| **Education** *(ref: No formal education)* | | | | |  |
| Primary school | 0.77 (0.77–0.77) | <0.001 | 0.71 (0.71–0.71) | <0.001 | <0.001 |
| Secondary school | 0.65 (0.64–0.65) | <0.001 | 0.59 (0.59–0.60) | <0.001 |  |
| High school | 0.51 (0.51–0.51) | <0.001 | 0.35 (0.35–0.36) | <0.001 |  |
| Higher education | 0.45 (0.45–0.46) | <0.001 | 0.34 (0.34–0.34) | <0.001 |  |
| **Social security** (No vs Yes) | 1.26 (1.25–1.26) | <0.001 | 1.20 (1.20–1.21) | <0.001 | <0.001 |
| ***Living arrangement*** | | | | |  |
| **Living alone** (Yes vs *No*) | 1.09 (1.08–1.09) | <0.001 | 0.96 (0.95–0.96) | <0.001 | <0.001 |
| **Household size** (per person) | 1.04 (1.04–1.04) | <0.001 | 1.04 (1.04–1.04) | <0.001 | <0.001 |
| ***Health status*** | | | | |  |
| **Chronic disease** (Yes vs *No*) | 1.55 (1.55–1.55) | <0.001 | 1.47 (1.46–1.47) | <0.001 | <0.001 |
| **Self-rated health** *(ref: Very good)* | |  | | |  |
| Good | 1.93 (1.91–1.95) | <0.001 | 1.38 (1.35–1.41) | <0.001 | <0.001 |
| Fair | 4.77 (4.73–4.82) | <0.001 | 7.14 (6.97–7.31) | <0.001 |  |
| Poor | 8.80 (8.71–8.89) | <0.001 | 42.95 (41.93–43.98) | <0.001 |  |
| Very poor | 5.70 (5.61–5.80) | <0.001 | 87.58 (85.31–89.91) | <0.001 |  |
| **Physical activity** *(ref: 1–3 times monthly)* | | | | |  |
| Daily or almost daily | 0.61 (0.61–0.62) | <0.001 | 0.49 (0.49–0.50) | <0.001 | <0.001 |
| At least weekly | 0.83 (0.82–0.83) | <0.001 | 0.78 (0.77–0.79) | <0.001 |  |
| Rarely | 0.95 (0.94–0.95) | <0.001 | 1.27 (1.26–1.29) | <0.001 |  |
| Never | 1.12 (1.11–1.13) | <0.001 | 2.09 (2.07–2.11) | <0.001 |  |
| ***Environmental factors*** | | | | |  |
| **Elevator access** (Yes vs *No*) | 0.87 (0.87–0.87) | <0.001 | 0.89 (0.88–0.89) | <0.001 | <0.001 |
| **Internet access** (Yes vs *No*) | 0.87 (0.87–0.87) | <0.001 | 0.94 (0.93–0.94) | <0.001 | <0.001 |
| **Home care needs** (Yes vs *No*) | 0.90 (0.90–0.91) | <0.001 | 2.23 (2.22–2.24) | <0.001 | <0.001 |

For binary variables, the first category is compared against the second (reference). *p* (overall) represents a joint Wald test for the overall effect of each predictor across both outcome categories. OR, odds ratio; CI, confidence interval. Robust profile is the reference category. Models are estimated using nnet::multinom with sampling weights but without survey design correction for variance estimation. The extremely narrow CIs and uniformly significant p-values reflect model-based standard errors; therefore, statistical inference should rely on the survey-adjusted multinomial regression presented in Table 2 of the main manuscript. *n* = 26**,**905

Section 8: Measurement Invariance Assessment

Separate latent profile analyses were estimated for sex (males vs females) and age groups (50–64 vs ≥65 years) to investigate the robustness of the identified latent profile structure across key demographic strata.

**Sex-stratified analyses (S8)**

Among females (n = 14**,**259), the three-profile solution successfully converged with perfect classification accuracy (entropy = 1.00). The resulting profiles largely reflected those identified in the total sample, corresponding to robust, intermediate, and severe multisystem frailty patterns, with a clear gradient of increasing motor, sensory, cognitive, and functional impairment (Table S8).

By contrast, among males (n = 12**,**646), the three-profile solution did not converge under equal-variance specifications, and only the two-profile solution achieved stable estimation. Considering the absence of convergence, formal assessment of profile invariance across sex was not undertaken. Accordingly, latent profiles were defined in the pooled sample, and sex differences were investigated at the level of profile prevalence and associated characteristics rather than structural equivalence (Table S8).

**Age-stratified analyses (S8)**

Age-stratified analyses provided more robust support for structural consistency of the three-profile solution. Among adults aged ≥65 years (n = 10**,**348), the three-profile model converged with perfect classification accuracy (entropy = 1.00), and the profile indicator patterns strongly paralleled those observed in the total sample, again reflecting robust, intermediate, and severe multisystem frailty phenotypes (Table S8).

Among adults aged 50–64 years (n = 16**,**557), the three-profile solution also converged but with a lower classification certainty (entropy = 0.94) and a meaningfully different profile structure. Although a robust profile (n = 9701; 58.6%) was clearly identifiable, the remaining two profiles diverged from those in the total sample. One profile (n = 2374; 14.3%) demonstrated a sensory-dominant pattern characterised by elevated hearing difficulty (mean = 1.11) with relatively modest motor impairment (walking difficulty mean = 0.79). The other profile (n = 4482; 27.1%) exhibited moderate motor impairment (walking difficulty mean = 1.23) without the pronounced multisystem deficits observed in the severe frailty profile of the total sample (walking difficulty mean = 2.06). Collectively, these results suggest that the severe multisystem frailty phenotype, defined by substantial impairment across motor, sensory, cognitive, and functional domains, predominantly emerges among older adults, whereas younger adults are more prone to exhibit domain-specific or transitional impairment patterns (Table S8).

**Table S8. Comparison of three-class latent profile solutions across demographic subgroups**

| **Subgroup** | **n** | **Three-class solution converged** | **Entropy** | **Robust n (%)** | **Intermediate n (%)** | **Severe n (%)** |
| --- | --- | --- | --- | --- | --- | --- |
| Total sample | 26,905 | Yes | 1.00 | 13**,**556 (50.4) | 9270 (34.5) | 4079 (15.2) |
| Females | 14,259 | Yes | 1.00 | 5823 (40.8) | 5541 (38.9) | 2895 (20.3) |
| Males | 12,646 | No | - | Not estimable | Not estimable | Not estimable |
| Aged ≥ 65 years | 10,348 | Yes | 1.00 | 3439 (33.2) | 4327 (41.8) | 2582 (25.0) |
| Aged 50–64 years | 16,557 | Yes | 0.94 | 9701 (58.6) | 4482 (27.1)* | 2374 (14.3)* |

Percentages are based on unweighted sample counts. Among adults aged 50–64 years, the three-class solution converges but with a different profile structure. One class exhibits a sensory-dominant pattern characterised by elevated hearing difficulty (mean = 1.11) with relatively modest motor impairment (walking difficulty mean = 0.79), whereas another class shows moderate motor impairment (walking difficulty mean = 1.23) without the marked multisystem deficits characteristic of severe frailty in the total sample (walking difficulty mean = 2.06). These findings indicate that the severe multisystem frailty phenotype predominantly emerges in older age.

Overall, the three-profile frailty structure demonstrated configural invariance among females and older adults (≥65 years) but not among males and younger adults (50–64 years). The nonconvergence of the three-class model among males and the lack of a severe multisystem frailty phenotype among younger adults suggest that this frailty typology can be most applicable to populations with sufficiently progressed functional decline to produce distinct multi-domain impairment patterns. These findings highlight the significance of age- and potentially sex-specific approaches to frailty assessment.

Section 9: Subgroup-stratified Multinomial Regression Analyses

Stratified survey-weighted multinomial logistic regression models were estimated to investigate whether associations between predictors and multisystem frailty profiles varied by sex and age groups.

**Sex-stratified analyses:** Among males (n = 12**,**646), the lack of social security coverage was more strongly associated with intermediate (odds ratio [OR], 1.50; 95% CI, 1.27–1.77) and severe (OR, 1.59; 95% CI, 1.23–2.06) frailties than among females, in whom no statistically significant association was observed (intermediate: OR, 1.11; p = 0.128; severe: OR, 1.04; p = 0.681). Physical inactivity (“never”) was associated with severe frailty in both sexes; however, males showed a larger magnitude of association (OR, 2.97; 95% CI, 1.76–5.00) than females (OR, 1.86; 95% CI, 1.33–2.60). Access to a building elevator revealed a protective association only among females, being associated with lower odds of intermediate (OR, 0.81) and severe (OR, 0.83) frailties, whereas males exhibited no significant association. Educational attainment demonstrated a steeper protective gradient among females, with higher education showing a stronger inverse association with severe frailty than that among males (OR, 0.31 vs 0.39) (Table S9).

**Table S9. Sex-stratified survey-weighted multinomial logistic regression**

| **Variable** | **Males (n = 12 646)** | | **Females (n = 14 259)** | |
| --- | --- | --- | --- | --- |
|  | **Intermediate**  **OR (95% CI)** | **Severe**  **OR (95% CI)** | **Intermediate**  **OR (95% CI)** | **Severe**  **OR (95% CI)** |
| ***Demographics*** | | | | |
| **Age (per year)** | 1.06 (1.05–1.06)*** | 1.09 (1.08–1.10)*** | 1.04 (1.03–1.05)*** | 1.07 (1.06–1.08)*** |
| **Marital status** *(ref: Divorced/separated)* | | | | |
| Widowed | 1.18 (0.86–1.62) | 1.48 (0.85–2.58) | 1.20 (0.96–1.50) | 1.21 (0.88–1.65) |
| Married | 1.24 (0.93–1.66) | 1.19 (0.70–2.01) | 1.08 (0.88–1.34) | 0.96 (0.71–1.29) |
| Never married | 0.66 (0.42–1.02) | 0.82 (0.37–1.81) | 0.83 (0.59–1.15) | 0.84 (0.51–1.39) |
| ***Socioeconomic*** | | | | |
| **Education** *(ref: No formal)* | | | | |
| Primary school | 0.73 (0.59–0.90)** | 0.75 (0.56–1.00) | 0.79 (0.70–0.89)*** | 0.71 (0.61–0.82)*** |
| Middle school | 0.63 (0.49–0.81)*** | 0.67 (0.45–1.00)* | 0.65 (0.53–0.80)*** | 0.56 (0.41–0.77)*** |
| High school | 0.52 (0.41–0.67)*** | 0.47 (0.32–0.70)*** | 0.48 (0.40–0.58)*** | 0.28 (0.21–0.38)*** |
| Higher education | 0.47 (0.36–0.60)*** | 0.39 (0.25–0.59)*** | 0.40 (0.32–0.50)*** | 0.31 (0.22–0.43)*** |
| **Social security**  (No vs *Yes*) | 1.50 (1.27–1.77)*** | 1.59 (1.23–2.06)*** | 1.11 (0.97–1.27) | 1.04 (0.87–1.23) |
| ***Living arrangement*** | | | | |
| **Living alone**  (Yes vs *No*) | 1.01 (0.80–1.28) | 0.89 (0.60–1.30) | 1.18 (1.00–1.41) | 1.03 (0.82–1.28) |
| **Household size**  (per person) | 1.04 (1.01–1.08)* | 1.05 (0.99–1.12) | 1.04 (1.00–1.08)* | 1.04 (0.99–1.09) |
| ***Health status*** | | | | |
| **Chronic disease**  (Yes vs *No*) | 1.53 (1.36–1.71)*** | 1.73 (1.35–2.21)*** | 1.55 (1.39–1.73)*** | 1.33 (1.12–1.58)** |
| **Self-rated health** *(ref: Very good)* | | | | |
| Good | 2.14 (1.41–3.25)*** | 0.72 (0.31–1.69) | 1.72 (1.09–2.74)* | 2.81 (1.07–7.37)* |
| Fair | 5.54 (3.65–8.42)*** | 3.46 (1.51–7.92)** | 4.12 (2.59–6.55)*** | 15.0 (5.79–39.1)*** |
| Poor | 12.1 (7.81–18.9)*** | 25.9 (11.2–59.9)*** | 6.41 (3.96–10.4)*** | 75.7 (28.9–198)*** |
| Very poor | 9.10 (4.41–18.8)*** | 78.7 (29.5–210)*** | 3.39 (1.76–6.53)*** | 109 (38.8–309)*** |
| **Physical activity** *(ref: Monthly)* | | | | |
| Daily | 0.65 (0.51–0.82)*** | 0.72 (0.41–1.26) | 0.57 (0.46–0.72)*** | 0.41 (0.28–0.61)*** |
| Weekly | 0.91 (0.71–1.17) | 1.21 (0.67–2.20) | 0.76 (0.60–0.95)* | 0.63 (0.43–0.93)* |
| Rarely | 0.92 (0.72–1.17) | 1.71 (0.99–2.95) | 0.96 (0.77–1.20) | 1.15 (0.81–1.63) |
| Never | 1.07 (0.85–1.34) | 2.97 (1.76–5.00)*** | 1.16 (0.94–1.44) | 1.86 (1.33–2.60)*** |
| ***Environmental*** | | | | |
| **Elevator access**  (Yes vs *No*) | 0.94 (0.84–1.06) | 0.99 (0.80–1.22) | 0.81 (0.73–0.90)*** | 0.83 (0.71–0.96)* |
| **Internet access**  (Yes vs *No*) | 0.87 (0.78–0.97)* | 0.96 (0.79–1.16) | 0.88 (0.79–0.97)* | 0.93 (0.81–1.07) |
| **Home care needs**  (Yes vs *No*) | 0.98 (0.80–1.20) | 2.48 (1.94–3.18)*** | 0.86 (0.71–1.03) | 2.09 (1.73–2.53)*** |

*p < 0.05, **p < 0.01, ***p < 0.001. Survey-weighted multinomial logistic regression with the robust profile as the reference category. For binary variables, the first category is compared against the second (reference). Sex is excluded from the stratified models as it is the stratification variable. OR, odds ratio; CI, confidence interval. McFadden pseudo-R²: males = 0.218; females = 0.210

**Age-stratified analyses:** Survey-weighted multinomial logistic regression models were separately re-estimated for individuals aged 50–64 years and those aged ≥65 years to explore whether associations between predictors and multisystem frailty profiles differed by age (Table S10).

**Table S10. Age-stratified survey-weighted multinomial logistic regression**

| **Variable** | **50–64 years (n = 16 557)** | | **≥ 65 years (n = 10 348)** | |
| --- | --- | --- | --- | --- |
|  | **Intermediate**  **OR (95% CI)** | **Severe**  **OR (95% CI)** | **Intermediate**  **OR (95% CI)** | **Severe**  **OR (95% CI)** |
| ***Demographics*** | | | | |
| **Age (per year)** | 1.04 (1.03–1.05)*** | 1.07 (1.06–1.09)*** | 1.06 (1.04–1.07)*** | 1.09 (1.08–1.11)*** |
| **Sex**  (Females vs *Males*) | 1.64 (1.50–1.79)*** | 2.21 (1.89–2.58)*** | 1.49 (1.32–1.68)*** | 2.25 (1.91–2.64)*** |
| **Marital status** *(ref: Divorced/separated)* | | | | |
| Widowed | 1.22 (0.97–1.54) | 1.84 (1.27–2.67)** | 1.29 (0.95–1.76) | 1.08 (0.71–1.64) |
| Married | 1.10 (0.90–1.35) | 1.22 (0.87–1.71) | 1.27 (0.92–1.75) | 0.96 (0.62–1.48) |
| Never married | 0.68 (0.50–0.93)* | 0.90 (0.53–1.53) | 1.09 (0.65–1.84) | 0.89 (0.43–1.83) |
| ***Socioeconomic*** | | | | |
| **Education** *(ref: No formal)* | | | | |
| Primary school | 0.75 (0.66–0.86)*** | 0.65 (0.53–0.80)*** | 0.79 (0.68–0.91)** | 0.75 (0.63–0.90)** |
| Middle school | 0.61 (0.50–0.74)*** | 0.51 (0.37–0.70)*** | 0.71 (0.56–0.91)** | 0.71 (0.50–1.01) |
| High school | 0.46 (0.39–0.56)*** | 0.30 (0.22–0.41)*** | 0.62 (0.50–0.78)*** | 0.44 (0.31–0.60)*** |
| Higher education | 0.40 (0.33–0.49)*** | 0.28 (0.19–0.40)*** | 0.55 (0.44–0.69)*** | 0.44 (0.32–0.62)*** |
| **Social security**  (No vs *Yes*) | 1.23 (1.08–1.40)** | 1.15 (0.94–1.40) | 1.29 (1.06–1.57)** | 1.25 (1.00–1.57) |
| ***Living arrangement*** | | | | |
| **Living alone**  (Yes vs *No*) | 1.00 (0.83–1.21) | 1.15 (0.85–1.55) | 1.13 (0.92–1.39) | 0.89 (0.69–1.15) |
| **Household size**  (per person) | 1.04 (1.01–1.08)** | 1.04 (0.99–1.10) | 1.03 (0.99–1.08) | 1.04 (0.98–1.09) |
| ***Health status*** | | | | |
| **Chronic disease**  (Yes vs No) | 1.52 (1.38–1.68)*** | 1.41 (1.16–1.71)*** | 1.58 (1.38–1.81)*** | 1.48 (1.21–1.82)*** |
| **Self-rated health** *(ref: Very good)* | | | | |
| Good | 1.60 (1.13–2.28)** | 5.02 (0.69–36.5) | 3.08 (1.75–5.45)*** | 0.93 (0.42–2.08) |
| Fair | 4.22 (2.95–6.03)*** | 33.2 (4.62–239)*** | 6.79 (3.85–12.0)*** | 3.82 (1.74–8.36)*** |
| Poor | 8.16 (5.59–11.9)*** | 220 (30.5–1590)*** | 11.4 (6.34–20.5)*** | 20.2 (9.12–44.8)*** |
| Very poor | 5.14 (2.78–9.52)*** | 496 (65.7–3740)*** | 7.00 (3.15–15.6)*** | 34.2 (13.4–87.6)*** |
| **Physical activity** *(ref: Monthly)* | | | | |
| Daily | 0.60 (0.50–0.74)*** | 0.44 (0.30–0.64)*** | 0.65 (0.49–0.86)** | 0.59 (0.37–0.96)* |
| Weekly | 0.76 (0.62–0.94)** | 0.64 (0.43–0.95)* | 0.98 (0.73–1.31) | 1.08 (0.66–1.78) |
| Rarely | 0.87 (0.71–1.06) | 0.91 (0.64–1.30) | 1.14 (0.86–1.51) | 2.03 (1.28–3.21)** |
| Never | 1.01 (0.83–1.21) | 1.40 (0.99–1.96) | 1.42 (1.09–1.85)* | 3.56 (2.29–5.54)*** |
| ***Environmental*** | | | | |
| **Elevator access**  (Yes vs *No*) | 0.89 (0.81–0.98)* | 0.88 (0.74–1.05) | 0.83 (0.73–0.94)** | 0.87 (0.73–1.04) |
| **Internet access**  (Yes vs *No*) | 0.89 (0.81–0.98)* | 1.03 (0.88–1.21) | 0.82 (0.73–0.93)** | 0.83 (0.70–0.98)* |
| **Home care needs**  (Yes vs *No*) | 0.83 (0.69–1.00)* | 1.89 (1.51–2.37)*** | 1.02 (0.83–1.26) | 2.62 (2.10–3.26)*** |

*p < 0.05, **p < 0.01, ***p < 0.001. Survey-weighted multinomial logistic regression with the robust profile as the reference category. For binary variables, the first category is compared against the second (reference). Age is retained as a continuous covariate to adjust for within-group age variation. OR, odds ratio; CI, confidence interval. McFadden pseudo-R²: 50–64 years = 0.187; ≥65 years = 0.210.

The effect of female sex on frailty risk was consistent across age groups, with females exhibiting approximately twofold higher severe frailty odds in the 50–64- (OR, 2.21; 95% CI, 1.89–2.58) and ≥65-year-old age groups (OR, 2.25; 95% CI, 1.91–2.64). By contrast, older adults demonstrated a substantially stronger association between physical inactivity and severe frailty, with those aged ≥65 years showing markedly elevated odds (OR, 3.56; 95% CI, 2.29–5.54) than those aged 50–64 years (OR, 1.40; 95% CI, 0.99–1.96; p = 0.054). Self-rated health demonstrated a pronounced age interaction. Very poor self-rated health was associated with extremely high odds of severe frailty among adults aged 50–64 years (OR, 496; 95% CI, 66–3740), compared with a substantially lower, although still strong, association among those aged ≥65 years (OR, 34.2; 95% CI, 13.4–87.6). This trend suggests that self-reported very poor health at younger ages can reflect a more severe underlying pathology or advanced multisystem impairment. Widowhood was significantly associated with severe frailty only in the 50–64-year-old age group (OR, 1.84; 95% CI, 1.27–2.67) (Table S10).

Section 10: Hierarchical Multinomial Logistic Regression with Nested Predictor Blocks

**Rationale for hierarchical block entry**

We employed a hierarchical multinomial logistic regression strategy to investigate the relative contribution of distinct predictor domains to multisystem frailty profile membership. This approach enters predictors in sequentially nested blocks, ordered according to a theoretical framework that moves from distal (demographic) to proximal (health-related and contextual) factors. Each successive model encompasses all variables from preceding blocks, facilitating incremental explanatory contribution assessment through changes in model fit indices.

This strategy varies from standard multivariable regression, which simultaneously estimates all predictors without distinguishing their relative significance. By contrast, hierarchical block entry quantifies how much additional explanatory contribution each domain provides beyond previously entered factors. This is particularly valuable for identifying modifiable targets: when health behaviours explain a substantial incremental contribution after controlling for demographics and socioeconomic position, interventions targeting these behaviours may reduce frailty burden independent of structural disadvantage.

**Multilevel structure assessment**

The survey design included individuals nested within households, which could motivate multilevel (mixed-effects) modeling with random intercepts for households. To evaluate this study design, we estimated intraclass correlation coefficients (ICCs) from null random-intercept logistic regression models at both household and regional levels (Table S11).

**Table S11. Assessment of clustering structure for consideration of multilevel modelling**

| **Level** | **Number of clusters** | **Mean cluster size** | **Single-unit clusters (%)** | **ICC**  **(null model)** | **ICC**  **(conditional model)** | **DEFF** |
| --- | --- | --- | --- | --- | --- | --- |
| Household | 18 115 | 1.48 | 53.4 | 0.188 | 0.197 | 1.09 |
| Region (NUTS-1) | 12 | 2 242 | — | 0.016 | — | — |

Intraclass correlation coefficients (ICCs) are estimated from unweighted random-intercept logistic models contrasting robust versus non-robust frailty profiles. Conditional ICCs are obtained following adjusting for selected individual-level covariates (age, sex, education, chronic disease, and self-rated health). The design effect (DEFF) is calculated as 1 + (m − 1) × ICC₁, where *m* denotes the mean cluster size. Dashes indicate quantities not applicable or not estimated. NUTS-1 refers to the first level of the Nomenclature of Territorial Units for Statistics.

At the household level, the null model ICC was 0.188, indicating that between-household differences accounted for approximately 19% of the variance in frailty profile membership. Although this finding reflected moderate clustering, its practical impact on statistical inference was minimal for three reasons. First, >50% of households (53.4%) included only one eligible respondent aged ≥50 years. Second, the mean household cluster size was small (1.48 persons). Third, the resulting design effect was close to unity (DEFF = 1.09), implying that treating observations as independent would inflate standard errors by <5%. After adjusting for individual-level covariates, the conditional ICC remained similar (0.197), indicating persistence of household-level variance but continued negligible impact on variance estimation owing to the small cluster sizes.

At the regional (Nomenclature of Territorial Units for Statistics, Level 1 [NUTS-1]) level, the ICC was 0.016, indicating that regional differences accounted for <2% of the variance in frailty profile membership. This value falls well below the 0.05 conventional threshold for meaningful clustering. Moreover, with only 12 regions, the number of higher-level units was inadequate for reliable estimation of random effects; simulation studies have suggested that stable variance component estimation require at least 30 clusters (Hox et al. 2017).

Based on these findings, we adopted a single-level survey-weighted approach rather than multilevel modelling. Four considerations supported this decision: (1) the limited effective clustering at the household level (DEFF, approximately 1); (2) the deficient number of regional units for dependable random effects estimation; (3) the analytic objective of quantifying incremental predictor block contributions rather than decomposing variance across levels; and (4) the greater compatibility of complex survey weights with design-based approaches than that of likelihood-based multilevel models for multinomial outcomes. We included region as a fixed effect in the final model block, which captures regional disparities without imposing distributional assumptions required for random effects. This approach does not preclude future multilevel analyses investigating household- or regional-level variance components.

**Model specification**

We estimated five sequentially nested multinomial logistic regression models with the robust profile as the reference category. Let $Y_{i}\in\left\{ Robust, Intermediate, Severe \right\}$ denote the frailty profile for individual $i$. The membership probability in profile $k$ relative to the robust profile is modelled as:

$$\log\left( \frac{P\left( Y_{i}=k \right)}{P\left( Y_{i}=Robust \right)} \right)=\beta_{0k}+\mathbb{X}_{i}^{\left( m \right)}\mathcal{B}_{k}^{\left( m \right)}$$

where $\mathbb{X}_{i}^{\left( m \right)}$ indicates the predictor matrix for model $m$ and $k\in\left\{ Intermediate, Severe \right\}$. The five models were specified as follows:

- Model 1 (Demographics):

$$\mathbb{X}^{\left( 1 \right)}=\left\{ Age, Sex, Marital Status \right\}.$$

- Model 2 (+ Health and Lifestyle):

$$\mathbb{X}^{\left( 2 \right)}=\mathbb{X}^{\left( 1 \right)}\cup\left\{ Chronic disease,Self-rated health,Physical activity frequency,Living alone \right\}.$$

- Model 3 (+ Socioeconomic):

$$\mathbb{X}^{\left( 3 \right)}=\mathbb{X}^{\left( 2 \right)}\cup\left\{ Education,Social security \right\}.$$

- Model 4 (+ Environmental/Household):

$$\mathbb{X}^{\left( 4 \right)}=\mathbb{X}^{\left( 3 \right)}\cup\left\{ Elevator access,Internet access,Home care need,Household size \right\}.$$

- Model 5 (+ Regional context):

$$\mathbb{X}^{\left( 5 \right)}=\mathbb{X}^{\left( 4 \right)}\cup\left\{ NUTS-1 region \right\}.$$

Model fit was assessed using McFadden pseudo-R², calculated as:

$$R_{McF}^{2}=1-\frac{\log L_{full}}{\log L_{null}}$$

where $\log L_{full}$ and $\log L_{null}$​ represent the log-likelihoods of the fitted and intercept-only models, respectively. The incremental contribution of each block was quantified as $\Delta R^{2}=R_{m}^{2}-R_{m-1}^{2}$.

The statistical significance of each block was evaluated using likelihood ratio tests comparing nested models:

$$LRT=-2(\log L_{m-1}-\log L_{m}\sim\chi_{df}^{2}$$

where $df$ equals the difference in parameters between models.

The following reference categories were employed for categorical predictors: male (sex), divorced/separated (marital status), no formal education (education), no (social security, living alone, chronic disease, elevator access, internet access, and home care needs), 1–3 times monthly (physical activity), very good (self-rated health), and Istanbul (NUTS-1 region). Istanbul, being the largest metropolitan area with the most comprehensive healthcare infrastructure, was selected as the regional reference.

Several predictors contain multiple categories representing ordinal or nominal distinctions: marital status (4 categories), education (5 categories), self-rated health (5 categories), physical activity frequency (5 categories), and NUTS-1 region (12 categories). Instead of collapsing these categories into binary indicators, we retained their full categorical structure to preserve information and facilitate the examination of dose–response patterns (for ordinal variables) or regional heterogeneity (for NUTS-1). This approach maintains fidelity to the underlying data structure while enabling each category’s contribution to be estimated.

The full model (Model 5) revealed distinct association patterns across predictor domains, with health status emerging as the factor most strongly associated with frailty profile membership. Self-rated health exhibited a striking gradient: compared with those reporting very good health, individuals rating their health as poor had ORs of 9.10 and 47.89 for intermediate and severe frailties, respectively, whereas those reporting very poor health demonstrated even more pronounced associations with severe frailty (OR, 95.78). The intermediate profile showed a paradoxical pattern wherein very poor health was associated with lower odds (OR, 5.87) than poor health (OR, 9.10), suggesting that individuals with the worst subjective health status directly shift to severe multisystem impairment instead of intermediate states. E-values for self-rated health associations were >90, indicating that these findings are highly robust to potential unmeasured confounding.

Regarding severe profile membership, those reporting very poor health demonstrated even more pronounced associations with severe frailty (OR, 95.78; 95% CI, 45.67–200.88), representing approximately a 100-fold increase in odds. The presence of chronic disease approximately doubled the odds of non-robust classification (OR, 1.55 for intermediate; OR, 1.49 for severe). Demographic factors established a foundational risk gradient: females experienced substantially elevated odds of intermediate (OR, 1.60) and severe (OR = 2.27) frailties compared with males, and each additional year of age increased the odds by 5% and 8% for intermediate and severe profile membership, respectively. Education revealed a clear protective dose–response relationship, with higher education levels associated with 54% lower odds of intermediate (OR, 0.46) and 65% lower odds of severe (OR, 0.35) frailties than no formal education; this gradient was consistent across all education levels, suggesting a graded inverse association between educational attainment and multisystem frailty. Physical activity frequency showed asymmetric effects across profiles: daily or near-daily activity was protective for intermediate (OR, 0.61) and severe (OR, 0.47) frailties, whereas physical inactivity was associated with significantly increased odds of severe frailty only (OR, 2.08), suggesting that sedentary behaviour is more strongly associated with advanced frailty states than with overall frailty risk. Environmental accessibility factors exhibited modest but significant associations, with elevator access (OR, 0.86 for both profiles) and internet access (OR, 0.89 for intermediate) showing inverse associations, potentially reflecting both functional accessibility and broader indicators of socioeconomic resources; notably, home care needs showed divergent associations, with no significant effect on intermediate frailty (OR, 0.91; p = 0.187) but was strongly positively associated with severe frailty (OR, 2.26), consistent with home care primarily operating as a consequence of advanced functional decline rather than an upstream factor. Regional disparities persisted even after adjusting for all individual-level factors: compared with residents of Istanbul, those of Mediterranean (OR, 1.26 for intermediate; OR, 1.64 for severe) and Central Anatolia (OR, 1.42 for intermediate; OR, 1.32 for severe) exhibited increased frailty risk, whereas those of Aegean (OR, 0.59 for severe), Eastern Black Sea (OR, 0.65–0.69 for both profiles), and Western Marmara (OR, 0.65 for severe) exhibited lower risk, suggesting that geographic context captures unmeasured environmental, healthcare access, or cultural factors associated with frailty beyond individual characteristics. Living alone was included in the Health and Lifestyle block based on its conceptualisation as a social health risk factor. Household size, categorized with Environmental/Household factors, showed statistical significance for intermediate frailty (OR, 1.04; p = 0.006) but minimal practical impact, consistent with the modest incremental R² contribution (2.2%) observed for this block in the hierarchical analysis. Marital status associations were modest and mainly nonsignificant in the fully adjusted model, with only widowhood showing a borderline increase for intermediate frailty (OR, 1.22; p = 0.026), suggesting that age and sex predominantly confound the observed bivariate associations between marital status and frailty. Collectively, these findings identify self-rated health, chronic disease presence, female sex, advancing age, and physical inactivity as the strongest risk factors for multisystem frailty, whereas higher education level and regular physical activity emerge as potentially modifiable factors inversely associated with frailty; the persistence of regional disparities after comprehensive adjustment highlights the relevance of place-based approaches to frailty prevention that consider contextual factors beyond individual-level characteristics (Table S12).

**Table S12. Full model results from hierarchical multinomial logistic regression (Model 5) for multisystem frailty profiles**

| **Variable** | **Intermediate vs Robust** | | **Severe vs Robust** | |
| --- | --- | --- | --- | --- |
|  | **OR (95% CI)** | **p** | **OR (95% CI)** | **p** |
| **Block 1: Demographics** | | | | |
| Age (per year) | 1.05 (1.04–1.05) | <0.001 | 1.08 (1.07–1.09) | <0.001 |
| Sex *(Females vs Males)* | 1.60 (1.49–1.72) | <0.001 | 2.27 (2.03–2.53) | <0.001 |
| Marital status *(ref: Divorced/separated)* |  |  |  |  |
| Widowed | 1.22 (1.02–1.46) | 0.026 | 1.30 (0.99–1.70) | 0.062 |
| Married | 1.16 (0.98–1.37) | 0.087 | 1.05 (0.81–1.37) | 0.714 |
| Never married | 0.77 (0.59–1.00) | 0.052 | 0.84 (0.55–1.29) | 0.435 |
| **Block 2: Health and Lifestyle** | | | | |
| Chronic disease *(Yes vs No)* | 1.55 (1.43–1.68) | <0.001 | 1.49 (1.30–1.72) | <0.001 |
| Self-rated health *(ref: Very good)* |  |  |  |  |
| Good | 1.94 (1.42–2.65) | <0.001 | 1.42 (0.73–2.76) | 0.302 |
| Fair | 4.81 (3.52–6.58) | <0.001 | 7.47 (3.88–14.38) | <0.001 |
| Poor | 9.10 (6.56–12.63) | <0.001 | 47.89 (24.72–92.80) | <0.001 |
| Very poor | 5.90 (3.62–9.61) | <0.001 | 95.78 (45.67–200.88) | <0.001 |
| Physical activity *(ref: 1–3 times monthly)* |  |  |  |  |
| Daily or almost daily | 0.61 (0.52–0.72) | <0.001 | 0.47 (0.34–0.63) | <0.001 |
| At least weekly | 0.81 (0.69–0.96) | 0.018 | 0.74 (0.54–1.02) | 0.067 |
| Rarely | 0.94 (0.80–1.11) | 0.492 | 1.24 (0.93–1.66) | 0.145 |
| Never | 1.10 (0.94–1.29) | 0.217 | 2.08 (1.58–2.75) | <0.001 |
| Living alone *(Yes vs No)* | 1.09 (0.95–1.25) | 0.220 | 0.96 (0.80–1.16) | 0.681 |
| **Block 3: Socioeconomic** | | | | |
| Education *(ref: No formal education)* |  |  |  |  |
| Primary school | 0.78 (0.70–0.86) | <0.001 | 0.73 (0.63–0.83) | <0.001 |
| Middle school | 0.65 (0.56–0.75) | <0.001 | 0.60 (0.48–0.76) | <0.001 |
| High school | 0.52 (0.45–0.59) | <0.001 | 0.37 (0.29–0.46) | <0.001 |
| Higher education | 0.46 (0.40–0.54) | <0.001 | 0.35 (0.27–0.46) | <0.001 |
| Social security *(No vs Yes)* | 1.22 (1.10–1.37) | <0.001 | 1.15 (1.00–1.33) | 0.054 |
| **Block 4: Environmental/Household** | | | | |
| Elevator access *(Yes vs No)* | 0.86 (0.79–0.93) | <0.001 | 0.86 (0.76–0.97) | 0.016 |
| Internet access *(Yes vs No)* | 0.89 (0.82–0.95) | 0.001 | 0.93 (0.83–1.04) | 0.189 |
| Home care needs *(Yes vs No)* | 0.91 (0.80–1.05) | 0.187 | 2.26 (1.94–2.63) | <0.001 |
| Household size (per person) | 1.04 (1.01–1.07) | 0.006 | 1.04 (1.00–1.08) | 0.063 |
| **Block 5: Regional context** | | | | |
| Region *(ref: Istanbul)* |  |  |  |  |
| Mediterranean | 1.26 (1.11–1.44) | <0.001 | 1.64 (1.35–2.00) | <0.001 |
| West Anatolia | 0.98 (0.85–1.12) | 0.765 | 1.16 (0.94–1.42) | 0.164 |
| West Black Sea | 1.20 (1.04–1.37) | 0.012 | 1.21 (0.98–1.50) | 0.069 |
| West Marmara | 0.92 (0.79–1.06) | 0.236 | 0.65 (0.51–0.82) | <0.001 |
| East Black Sea | 0.69 (0.59–0.82) | <0.001 | 0.65 (0.50–0.83) | <0.001 |
| East Marmara | 1.14 (1.00–1.30) | 0.057 | 1.42 (1.16–1.74) | <0.001 |
| Aegean | 0.89 (0.79–1.00) | 0.057 | 0.59 (0.49–0.72) | <0.001 |
| Southeast Anatolia | 1.01 (0.86–1.19) | 0.894 | 1.03 (0.82–1.30) | 0.780 |
| Northeast Anatolia | 1.14 (0.96–1.36) | 0.135 | 0.96 (0.74–1.25) | 0.781 |
| Central Anatolia | 1.42 (1.22–1.66) | <0.001 | 1.32 (1.05–1.66) | 0.016 |
| Middle East Anatolia | 1.15 (0.97–1.36) | 0.097 | 0.74 (0.58–0.95) | 0.019 |

Survey-weighted multinomial logistic regression with the robust profile as the reference category. The model comprises all variables from blocks 1 to 5 entered sequentially; the incremental contribution of each block is presented in Table 3 of the main manuscript. Reference categories for categorical predictors include divorced/separated (marital status), no formal education (education), very good (self-rated health), 1–3 times monthly (physical activity), and Istanbul (NUTS-1 region). For binary variables, the first category listed is compared against the second (reference): females versus males (sex), yes versus no (chronic disease, living alone, elevator access, internet access, and home care needs), and no versus yes (social security). Age and household size are modelled as continuous variables. OR = odds ratio; CI = confidence interval. Final model McFadden pseudo-R² = 0.235. N = 26,905.

Section 11: Comparison with Conventional Frailty Classifications

To contextualise the LPA-derived multisystem frailty profiles against established frailty frameworks, two conventional classifications were constructed from the available survey data and compared with the latent profile solution: a modified Fried phenotype and a Rockwood-style Frailty Index.

The modified Fried phenotype was operationalised as a three-item proxy based on the closest available approximations to the original criteria proposed by Fried and colleagues (2001). Slowness was proxied by substantial walking difficulty (Washington Group item score ≥ 2, corresponding to "a lot of difficulty" or "cannot do at all"); weakness was proxied by substantial gripping difficulty (score ≥ 2); and low physical activity was defined as reporting no leisure-time physical activity ("never"). Participants meeting zero criteria were classified as robust, one criterion as pre-frail, and two or three criteria as frail. Two of the original five Fried criteria—unintentional weight loss and self-reported exhaustion—were not available in the survey instrument. This proxy therefore captures a subset of the physical phenotype and is expected to yield conservative prevalence estimates relative to the full five-item operationalisation.

The Frailty Index (FI) was constructed following the deficit accumulation approach described by Rockwood and Mitnitski (2007). Eleven health deficits were included: the eight LPA indicator variables (walking difficulty, gripping difficulty, falls, vision difficulty, hearing difficulty, learning difficulty, memory complaints, and ADL dependency) plus chronic disease presence, self-rated health, and physical inactivity. Ordinal items scored 0-3 were rescaled to 0-1 by dividing by 3; binary items (0-1) were retained as-is; self-rated health was mapped as very good = 0.00, good = 0.25, fair = 0.50, poor = 0.75, very poor = 1.00; and physical inactivity was mapped as daily/almost daily = 0.00, at least weekly = 0.25, 1-3 times monthly = 0.50, rarely = 0.75, never = 1.00. The FI was calculated as the arithmetic mean of all 11 deficit scores for each individual. Standard cutpoints were applied: FI < 0.10 (robust), 0.10-0.25 (pre-frail), and > 0.25 (frail). Although the recommended minimum number of deficits for constructing a Frailty Index is typically 30 or more (Searle et al. 2008), the present 11-item index was constructed for comparative purposes using the variables available within the survey instrument and is not intended as a standalone frailty assessment tool.

Survey-weighted prevalence estimates for the three classification approaches are presented in Table S13. The LPA multisystem approach and the modified Fried phenotype yielded broadly similar overall frailty prevalence estimates (48.6% vs 49.2% with any degree of frailty). In contrast, the Frailty Index classified a substantially larger proportion of the population as having some degree of frailty (87.9%), with 53.7% categorised as frail. This divergence reflects the broader scope of the deficit accumulation approach, which incorporates highly prevalent conditions such as chronic disease (64.7% of the sample), suboptimal self-rated health, and physical inactivity, resulting in elevated deficit counts even among individuals with limited functional impairment.

**Table S13. Comparison of frailty classification approaches: survey-weighted prevalence and cross-classification**

| ***Panel A. Survey-weighted prevalence by classification approach*** | | | |
| --- | --- | --- | --- |
| **Approach** | **Robust, % (95% CI)** | **Pre-frail/Intermediate, % (95% CI)** | **Frail/Severe, % (95% CI)** |
| LPA Multisystem | 51.4 (50.8-52.1) | 33.7 (33.1-34.3) | 14.8 (14.4-15.3) |
| Modified Fried (3-item) | 50.8 (50.2-51.5) | 33.3 (32.7-34.0) | 15.9 (15.4-16.3) |
| Frailty Index (11-item) | 12.1 (11.7-12.6) | 34.2 (33.6-34.8) | 53.7 (53.0-54.3) |
| ***Panel B. Cross-classification of LPA profiles against modified Fried phenotype, n (row %)*** | | | |
| **LPA Profile** | **Fried Robust** | **Fried Pre-frail** | **Fried Frail** |
| Robust | 8,935 (65.9%) | 4,525 (33.4%) | 96 (0.7%) |
| Intermediate | 4,441 (47.9%) | 4,366 (47.1%) | 463 (5.0%) |
| Severe | 0 (0.0%) | 278 (6.8%) | 3,801 (93.2%) |
| ***Panel C. Cross-classification of LPA profiles against Frailty Index, n (row %)*** | | | |
| **LPA Profile** | **FI Robust** | **FI Pre-frail** | **FI Frail** |
| Robust | 2,979 (22.0%) | 7,109 (52.4%) | 3,468 (25.6%) |
| Intermediate | 101 (1.1%) | 1,827 (19.7%) | 7,342 (79.2%) |
| Severe | 1 (0.0%) | 68 (1.7%) | 4,010 (98.3%) |

Panel A presents survey-weighted prevalence estimates with 95% confidence intervals. Panels B and C present unweighted cross-tabulations with row percentages. LPA, latent profile analysis; FI, Frailty Index. Modified Fried phenotype criteria: walking difficulty ≥ 2 (slowness proxy), gripping difficulty ≥ 2 (weakness proxy), and no physical activity (inactivity); 0 criteria = robust, 1 = pre-frail, ≥ 2 = frail. FI cutpoints: < 0.10 robust, 0.10-0.25 pre-frail, > 0.25 frail.

Cross-classification of LPA profiles against the modified Fried phenotype revealed substantial discordance in the intermediate frailty range (Table S13, Panel B). Among individuals classified within the LPA Intermediate profile, 47.9% (n = 4,441) were categorised as robust by the modified Fried criteria. These discordant cases—individuals identified as exhibiting multisystem frailty by LPA but classified as non-frail by the physical phenotype approach—constituted 16.5% of the total analytic sample. In contrast, only 96 individuals (0.4%) were classified as frail by the modified Fried criteria but robust by LPA, indicating that the multisystem approach captured nearly all individuals identified by the physical phenotype plus a substantial additional subpopulation.

Examination of the domain-specific impairment profiles of the discordant subgroup (LPA Intermediate, Fried Robust; n = 4,441) revealed that these individuals exhibited low motor impairment (mean walking difficulty 1.0; mean gripping difficulty 0.84)— below the modified Fried threshold of ≥ 2—but demonstrated notable sensory impairment (mean vision difficulty 0.59; mean hearing difficulty 0.31), cognitive complaints (42.1% reporting memory problems; mean learning difficulty 0.45), and functional limitations (8.1% ADL dependent). Domain composite scores for the discordant subgroup were 26.8% (motor), 15.0% (sensory), 28.6% (cognitive), and 8.1% (functional), confirming that these individuals were primarily characterised by sensory-cognitive vulnerability rather than motor impairment. For comparison, concordant severe/frail cases (identified by both approaches; n = 3,801) exhibited substantially higher impairment across all domains: motor 58.2%, sensory 24.8%, cognitive 47.4%, and functional 38.3%.

Agreement between classification approaches, assessed using Cohen's kappa, was moderate between LPA and the modified Fried phenotype (kappa = 0.400, observed agreement 63.6%) and poor between LPA and the Frailty Index (kappa = 0.096, observed agreement 32.8%). The low agreement with the FI reflects the fundamentally different classification logic of the deficit accumulation approach, which assigns frailty status based on the cumulative burden of health deficits without distinguishing clinically distinct impairment profiles.

These findings indicate that the LPA-derived multisystem profiles identify a subpopulation characterised by sensory and cognitive vulnerability that is not captured by physical phenotype-based criteria. The modified Fried proxy, which relies exclusively on motor and activity indicators, systematically classifies these individuals as robust despite measurable multidomain impairment. The deficit accumulation approach, while inclusive of non-physical domains, produces substantially higher prevalence estimates and does not differentiate between qualitatively distinct frailty phenotypes.

Section 12: Sensitivity Analysis for Unmeasured Confounding Using E-values

As associations may be distorted by unmeasured confounding, observational studies cannot establish causality. We calculated E-values for selected key predictors from the fully adjusted survey-weighted multinomial logistic regression model (Model 5) to quantify the robustness of the observed associations to potential unmeasured confounders.

The E-value represents the minimum strength of association on the risk ratio scale that an unmeasured confounder should have with both the exposure and the outcome, conditional on the measured covariates, to fully explain the observed association. Larger E-values indicate greater robustness to unmeasured confounding, whereas smaller values suggest that weaker confounding can attenuate the observed effect. For an estimated OR of >1, the E-value is defined as (VanderWeele and Ding 2017):

$$E-value=OR+\sqrt{OR\times\left( OR-1 \right)}.$$

For protective associations (OR < 1), the OR was inverted (1/OR) before calculation. E-values were computed for both the point estimates and the confidence interval bound closest to the null (lower bound for risk associations, upper bound for protective associations), following the approach proposed by VanderWeele and Ding (VanderWeele and Ding 2017).

**Table S14. E-values for sensitivity to unmeasured confounding in associations with severe multisystem frailty**

| **Variable** | **OR (95% CI)** | **E-value (point)** | **E-value (CI)** |
| --- | --- | --- | --- |
| Sex (Females vs *Males*) | 2.27 (2.03–2.53) | 3.97 | 3.48 |
| Age (per year) | 1.08 (1.07–1.09) | 1.37 | 1.34 |
| Education: Higher vs *None* | 0.35 (0.27–0.46) | 5.16 | 3.77 |
| Self-rated health: Poor vs *Very good* | 47.89 (24.72–92.80) | 95.28 | 48.93 |
| Self-rated health: Very poor vs *Very good* | 95.78 (45.67–200.88) | 191.06 | 90.84 |
| Chronic disease (Yes vs *No*) | 1.49 (1.30–1.72) | 2.34 | 1.92 |
| Physical activity: Daily vs *Monthly* | 0.47 (0.34–0.63) | 3.68 | 2.55 |
| Physical activity: Never vs *Monthly* | 2.08 (1.58–2.75) | 3.58 | 2.54 |
| Home care needs (Yes vs *No*) | 2.26 (1.94–2.63) | 3.95 | 3.29 |

E-values quantify the minimum strength of association on the risk ratio scale that an unmeasured confounder should have with both the exposure and the outcome, conditional on the measured covariates, to completely account for the observed association. E-values are calculated for point estimates and the confidence interval bound closest to the null. For protective associations (OR < 1), ORs are inverted before calculation. Larger E-values indicate greater robustness to potential unmeasured confounding.

The E-value analysis indicated that most key findings were robust to potential unmeasured confounding. Associations between self-rated health and severe multisystem frailty were exceptionally strong: an unmeasured confounder should be associated with both self-rated health and frailty by a factor of approximately 95 for poor health and 191 for very poor health to fully account for the observed association. These magnitudes considerably exceed the strength of known risk factors in ageing and frailty research, indicating a high degree of robustness. The protective association of higher education level (E-value = 5.16) and the associations with female sex, home care needs, and physical activity patterns (E-values ≈ 3.6–4.0) revealed strong robustness to unmeasured confounding. The association with chronic disease showed moderate robustness (E-value = 2.34), suggesting that although it is unlikely to be completely attributable to unmeasured confounding, some attenuation with further adjustment is probable. By contrast, the association with age exhibited limited robustness (E-value = 1.37), which is expected considering the modest effect size per 1-year increase; however, the cumulative effect of age over longer intervals would imply considerably greater robustness. Overall, these findings indicate that unmeasured confounding is unlikely to fully account for the principal conclusions regarding health status, education, sex, and physical activity, thereby reinforcing confidence in the observed associations despite the cross-sectional study design (Table S14).

Section 13: Statistical Software and Reproducibility

This study used data from the TURKSTAT Elderly Statistics Survey. The data are not publicly available owing to confidentiality restrictions imposed by TURKSTAT. Researchers wishing to access the data may submit a formal application through TURKSTAT’s official data access procedures.

All statistical analyses were performed using R version 4.4.2 (R Foundation for Statistical Computing, Vienna, Austria). Data management, statistical modelling, and visualisation were conducted using the packages listed in Table S15.

**Table S15. R packages used for statistical analysis**

| **Package** | **Version** | **Purpose** |
| --- | --- | --- |
| tidyLPA (Rosenberg et al. 2018) | 1.1.0 | Latent profile analysis |
| survey (Lumley 2024) | 4.4.2 | Survey-weighted descriptive statistics and design-based inference |
| svyVGAM (Lumley 2025) | 1.2.17 | Survey-weighted multinomial logistic regression |
| VGAM (Yee et al. 2015) | 1.1.12 | Vector generalized additive models |
| nnet (Venables and Ripley 2002) | 7.3–19 | Multinomial logistic regression (sensitivity analysis) |
| lme4 (Bates et al. 2015) | 1.1–35.5 | Mixed-effects models for ICC estimation |
| EValue (VanderWeele and Ding 2017) | 4.1.4 | E-value sensitivity analysis |
| tidyverse (Wickham et al. 2019) | 2.0.0 | Data manipulation and transformation |
| ggplot2 (Wickham 2016) | 4.0.0 | Data visualisation |
| patchwork (Pedersen 2024) | 1.3.0 | Combining multiple plots |
| ggtext (Claus and Brenton 2022) | 0.1.2 | Enhanced text rendering |
| gtsummary (Sjoberg et al. 2021) | 4.4.2 | Summary tables |
| psych (Revelle 2025) | 1.2.17 | Polychoric correlations |

LPA was performed using the tidyLPA package, which implements Gaussian finite mixture models via maximum likelihood estimation. Survey-weighted multinomial logistic regression models were estimated using the svyVGAM package (Lumley 2025), which extends the VGAM framework to accommodate complex survey designs. Taylor series linearisation was employed for computing design-based standard errors. ICCs used for assessing clustering at the household and regional levels were estimated using mixed-effects models fitted by restricted maximum likelihood via the lme4 package. Sensitivity to unmeasured confounding was evaluated using E-values, calculated using the EValue package following the methodology proposed by VanderWeele and Ding (VanderWeele and Ding 2017). A fixed random seed (set.seed(123)) was specified before all stochastic procedures, including LPA, to ensure reproducibility.

The analytical code supporting the findings of this study is available from the corresponding author upon reasonable request.

References for Supplementary Material

Bates D, Mächler M, Bolker B, Walker S (2015) Fitting Linear Mixed-Effects Models Using lme4. J Stat Softw 67:. https://doi.org/10.18637/jss.v067.i01

Claus OW, Brenton MW (2022) ggtext: Improved Text Rendering Support for “ggplot2”

Ertan T, Eker E (2000) Reliability, Validity, and Factor Structure of the Geriatric Depression Scale in Turkish Elderly: Are There Different Factor Structures for Different Cultures? Int Psychogeriatr 12:163–172. https://doi.org/10.1017/S1041610200006293

Fried LP, Tangen CM, Walston J, et al (2001) Frailty in Older Adults: Evidence for a Phenotype. J Gerontol A Biol Sci Med Sci 56:M146–M157. https://doi.org/10.1093/gerona/56.3.M146

Hox JJ, Moerbeek M, van de Schoot R (2017) Multilevel Analysis. Routledge, Third edition. | New York, NY : Routledge, 2017. |

Katz S, Ford AB, Moskowitz RW, et al (1963) Studies of Illness in the Aged. The Index Of ADL: A Standardized Measure of Biological and Psychosocial Function. JAMA 185:914–919. https://doi.org/10.1001/jama.1963.03060120024016

Lumley T (2024) survey: analysis of complex survey samples

Lumley T (2025) svyVGAM: Design-Based Inference in Vector Generalised Linear Models

Madans JH, Loeb ME, Altman BM (2011) Measuring disability and monitoring the UN Convention on the Rights of Persons with Disabilities: the work of the Washington Group on Disability Statistics. BMC Public Health 11:S4. https://doi.org/10.1186/1471-2458-11-S4-S4

Oberski D (2016) Mixture Models: Latent Profile and Latent Class Analysis. pp 275–287

Pedersen TL (2024) patchwork: The Composer of Plots

Revelle W (2025) psych: Procedures for Psychological, Psychometric, and Personality Research

Rockwood K, Mitnitski A (2007) Frailty in Relation to the Accumulation of Deficits. J Gerontol A Biol Sci Med Sci 62:722–727. https://doi.org/10.1093/gerona/62.7.722

Rosenberg J, Beymer P, Anderson D, et al (2018) tidyLPA: An R Package to Easily Carry Out Latent Profile Analysis (LPA) Using Open-Source or Commercial Software. J Open Source Softw 3:978. https://doi.org/10.21105/joss.00978

Searle SD, Mitnitski A, Gahbauer EA, et al (2008) A standard procedure for creating a frailty index. BMC Geriatr 8:24. https://doi.org/10.1186/1471-2318-8-24

Sjoberg DD, Whiting K, Curry M, et al (2021) Reproducible Summary Tables with the gtsummary Package. R J 13:570. https://doi.org/10.32614/RJ-2021-053

VanderWeele TJ, Ding P (2017) Sensitivity Analysis in Observational Research: Introducing the E-Value. Ann Intern Med 167:268–274. https://doi.org/10.7326/M16-2607

Venables WN, Ripley BD (2002) Modern Applied Statistics with S, MASS package in R. Springer New York, New York, NY

Wickham H (2016) Programming with ggplot2. In: Elegant Graphics for Data Analysis, 2nd edn. pp 241–253

Wickham H, Averick M, Bryan J, et al (2019) Welcome to the Tidyverse. J Open Source Softw 4:1686. https://doi.org/10.21105/joss.01686

Yee TW, Stoklosa J, Huggins RM (2015) The VGAM Package for Capture-Recapture Data Using the Conditional Likelihood. J Stat Softw 65:. https://doi.org/10.18637/jss.v065.i05

Yesavage JA, Brink TL, Rose TL, et al (1982) Development and validation of a geriatric depression screening scale: A preliminary report. J Psychiatr Res 17:37–49. https://doi.org/10.1016/0022-3956(82)90033-4
