# Supplementary material for: Multisystem frailty phenotypes and associated factors among older adults in Türkiye: A nationally representative study
Source: PLoS One. 2026 Jun 26;21(6):e0352767. doi: 10.1371/journal.pone.0352767 (PMC13308790; doi:10.1371/journal.pone.0352767)
Supplement: Attachment — Submitted filename: Revision Notes.docx [file pone.0352767.s003.docx]

Dear Dr. Pérez-Zepeda (Academic Editor) and Reviewer,

We would like to thank the Academic Editor and Reviewer #1 (Dr. Hiroshi Akasaka) for their thoughtful and constructive evaluation of our manuscript. We have carefully addressed each point raised, and we believe the revised manuscript has been substantially strengthened as a result. Below, we provide a point-by-point response to all comments. Reviewer/Editor comments are presented in bold, and our responses follow in regular text. All changes in the revised manuscript are highlighted using track changes.

**PART A: Journal Requirements (Academic Editor)**

**Journal Requirement 1: PLOS ONE Style and File Naming**

**Editor’s comment:** "Please ensure that your manuscript meets PLOS ONE’s style requirements, including those for file naming."

**Response:** We have carefully reviewed the PLOS ONE style templates and revised the manuscript to ensure full compliance with all formatting requirements. The manuscript is formatted with double spacing, continuous line numbers, and page numbers throughout. Heading levels are limited to three. All abbreviations are defined upon first appearance in the text. References follow Vancouver style with sequential numbering by order of first appearance. Figure captions are placed in the manuscript text immediately following the paragraph where each figure is first cited, and tables are similarly positioned after their first citation. Figure files are named according to PLOS ONE conventions (Fig1.tif, Fig2.tif), saved in TIFF format with LZW compression at 600 dpi, and uploaded as individual files separate from the manuscript. The manuscript now includes all required structural elements in the specified order: title page, abstract, introduction, methods, results, discussion, acknowledgments, references, and supporting information captions. The supporting information file has been renamed to conform to PLOS ONE naming conventions.

**Journal Requirement 2: Funding Information Mismatch**

**Editor’s comment:** "We note that the grant information you provided in the ‘Funding Information’ and ‘Financial Disclosure’ sections do not match. When you resubmit, please ensure that you provide the correct grant numbers for the awards you received for your study in the ‘Funding Information’ section."

**Response:** We apologise for the inconsistency between the Funding Information and Financial Disclosure sections in the original submission. This study was conducted within the scope of a project funded by the Scientific and Technological Research Council of Türkiye (TUBITAK) under the 1004 Centre of Excellence Support Program (Project No. 22AG017). However, as this is a secondary analysis of existing survey data, no direct financial resources from the grant were utilised in the conduct of this specific study. This distinction led to an inadvertent discrepancy between the two sections during the initial submission. Upon resubmission, we have ensured that both the Funding Information and Financial Disclosure fields in the Editorial Manager system contain consistent and accurate information, clearly stating the project affiliation while noting that the funder had no role in study design; data collection, analysis, or interpretation; writing of the manuscript; or the decision to submit for publication.

**Journal Requirement 3: Data Availability**

**Editor’s comment:** "All PLOS journals now require all data underlying the findings described in their manuscript to be freely available to other researchers, either (1) in a public repository, (2) within the manuscript itself, or (3) uploaded as supplementary information. This policy applies to all data except where public deposition would breach compliance with the protocol approved by your research ethics board. If your data cannot be made publicly available for ethical or legal reasons (e.g., public availability would compromise patient privacy), please explain your reasons on resubmission and your exemption request will be escalated for approval."

**Response:** The microdata used in this study are owned by the Turkish Statistical Institute (TURKSTAT), the official national statistical authority of the Republic of Türkiye, and were accessed under a formal data-use agreement that explicitly prohibits public redistribution or deposition of the individual-level dataset. This restriction is imposed by TURKSTAT as the data custodian and applies to all researchers who access the survey microdata, regardless of institutional affiliation. The dataset contains deidentified but potentially re-identifiable individual-level records from a nationally representative household survey, and TURKSTAT maintains exclusive control over data access to ensure compliance with national data protection regulations.

We respectfully request an exemption from the public data deposition requirement on these grounds. To ensure transparency and reproducibility despite this restriction, we have taken the following steps. First, all summary statistics, cross-tabulation tables, model coefficients, confidence intervals, and fit indices underlying the reported findings are presented in full in the manuscript tables and in the Supplementary Material (15 supplementary tables across 13 sections). Second, the analytic code used in this study is available from the corresponding author upon reasonable request. Third, researchers wishing to independently access the original microdata may submit a formal application through TURKSTAT's official data access procedures (contact: <https://www.tuik.gov.tr>). The data access process is clearly documented and routinely used by researchers in Türkiye and internationally.

We believe these provisions satisfy the spirit of the PLOS data availability policy by enabling full verification and replication of our findings while respecting the legal constraints imposed by the data custodian. We have updated the Data Availability Statement in the revised submission and the Data Sharing Statement in the title page to reflect this information.

**Journal Requirement 4: Reviewer-Recommended Citations**

**Editor’s comment:** "If the reviewer comments include a recommendation to cite specific previously published works, please review and evaluate these publications to determine whether they are relevant and should be cited. There is no requirement to cite these works unless the editor has indicated otherwise."

**Response:** The reviewer did not recommend any specific publications for citation. However, in addressing the reviewer's substantive comments—particularly regarding the comparison with conventional frailty classifications—we identified and incorporated several relevant references that strengthen the revised manuscript. These include studies on frailty classification discordance across instruments (Xue et al. 2020; Oviedo-Briones et al. 2021; Van der Elst et al. 2019) and foundational methodological references for the Frailty Index (Rockwood and Mitnitski 2007; Searle et al. 2008) and E-value sensitivity analysis (VanderWeele and Ding 2017). All newly added references were evaluated for relevance and cited only where they directly support the revised content.

**PART B: Reviewer #1 Comments (Dr. Hiroshi Akasaka)**

**General remark by Reviewer:** *"Using TURKSTAT data, the authors examined the prevalence of frailty and its associated factors among 26,905 older adults, representing approximately 20.2 million adults in Turkey, using a multidimensional index. According to this nationally representative analysis, 48.6% of adults aged 50 and older in Turkey exhibited some degree of frailty; this prevalence is significantly higher than estimates reported in many high-income countries and in previous studies conducted within Turkey. It is also noteworthy that the frailty profiles identified in this study spanned the domains of mobility, sensory function, cognition, and functional status. However, this paper has the following limitations."*

**Response:** We thank the reviewer for this positive and accurate summary of our work.

**Reviewer Comment 1: Comparative Analysis with Fried/Rockwood Criteria**

**Reviewer’s comment:** "The reviewer believes it is necessary to conduct a comparative analysis to determine how the prevalence of frailty changes when widely used standard criteria—such as Fried’s phenotypic criteria or Rockwood’s cumulative deficit criteria—are applied to the study population. The reviewer believes that this analysis will be particularly significant if it leads to the identification of populations previously slipped by conventional criteria or of phenotypes that could not be measured previously."

**Response:** We thank the reviewer for this important suggestion. To address this point, we conducted a comparative analysis by constructing two conventional frailty classifications from the available survey data and comparing them with the LPA-derived multisystem profiles.

A modified Fried phenotype was operationalised as a three-item proxy using the closest available approximations to the original criteria: substantial walking difficulty (Washington Group score ≥2) as a proxy for slowness, substantial gripping difficulty (score ≥2) as a proxy for weakness, and no leisure-time physical activity as a proxy for low activity. Two of the five original Fried criteria — unintentional weight loss and exhaustion — were not available in the survey instrument. A Rockwood-style Frailty Index was constructed using a deficit accumulation approach incorporating 11 health deficits rescaled to a 0–1 range, with standard cutpoints applied.

The results revealed notable differences in case identification across approaches. The modified Fried phenotype yielded a similar overall frailty prevalence (49.2%) to the LPA multisystem approach (48.6%), whereas the Frailty Index produced a substantially higher estimate (87.9%). Cross-classification of LPA profiles against the modified Fried phenotype demonstrated high concordance for the Severe profile (93.2% classified as Fried frail) but substantial discordance for the Intermediate profile, in which 47.9% of individuals (n = 4,441; 16.5% of the total sample) were classified as robust by the modified Fried criteria despite exhibiting multisystem impairment. These discordant individuals were characterised by sensory and cognitive vulnerability (mean vision difficulty 0.59; 42.1% memory complaints) with motor impairment below the Fried threshold, confirming that physical phenotype-based criteria systematically overlook individuals whose vulnerability manifests primarily in non-motor domains. Only 96 individuals (0.4%) showed the reverse pattern (Fried frail, LPA robust), indicating that the multisystem approach captured nearly all individuals identified by the physical phenotype plus a substantial additional subpopulation. Cohen's kappa between LPA and the modified Fried phenotype was 0.400, consistent with prior evidence of moderate agreement across frailty instruments.

These findings have been incorporated into the revised manuscript as follows: the methodology is described in the Statistical analysis subsection (Methods), the key results are reported in a new paragraph in the Results section, a detailed discussion contextualising these findings within the broader frailty classification literature has been added to the Discussion section (with supporting references from Xue et al. 2020, Oviedo-Briones et al. 2021, and Van der Elst et al. 2019), and the full cross-classification tables and domain-specific impairment profiles are presented in a new Supplementary Section 11 (Table S13). We believe this analysis directly addresses the reviewer's concern by demonstrating both how frailty prevalence changes under different classification approaches and, importantly, by identifying the specific subpopulation — characterised by sensory-cognitive vulnerability — that is captured by the multisystem approach but missed by conventional physical phenotype criteria.

**Reviewer Comment 2: Preventive and Improvement Strategies for Frailty**

**Reviewer’s comment:** "Based on the findings of this study, the reviewer believes it is necessary to discuss potential preventive and improvement strategies for frailty in the future."

**Response:** We thank the reviewer for highlighting this important aspect. The Discussion section has been substantially expanded to include a more detailed and concrete discussion of preventive and improvement strategies. Specifically, two new paragraphs have been added (replacing the previous brief mention) that address the following points.

First, building on the finding that frailty is increasingly recognised as a potentially reversible condition, we discuss how the identification of sensory-cognitive vulnerability as a distinguishing feature of the intermediate frailty profile has direct implications for screening design. We argue that screening strategies focused exclusively on physical function—as emphasised in traditional Fried-based approaches—risk overlooking a sizeable at-risk subpopulation, and that effective early detection requires multidomain assessment extending beyond motor performance to include routine vision and hearing evaluation, cognitive monitoring, and enquiry into subjective memory complaints.

Second, we discuss specific intervention strategies, including the growing evidence supporting multicomponent programmes that combine physical exercise with cognitive training, nutritional counselling, and social engagement. We highlight the strong protective association observed for daily physical activity in this study (OR 0.49 for severe frailty) as reinforcing the potential of community-based exercise initiatives as a scalable, low-cost preventive strategy. For Türkiye specifically, we discuss the potential for embedding multidomain frailty screening within the existing primary care infrastructure, including family health centres and home-based care programmes. We also note that the pronounced sex and age gradients in frailty prevalence suggest that prevention efforts should prioritise women and adults approaching the 65-year threshold. Finally, we discuss the broader applicability of combining self-reported, questionnaire-based indicators with data-driven profiling methods such as LPA as a practical pathway for population-level frailty surveillance in settings where performance-based clinical measures are not routinely available.

**PART C: Additional Items**

**C.1: ORCID Verification for Co-authors**

**Editor’s note:** *"Please encourage your coauthors to verify their ORCID iD within the submission system before final acceptance, as unverified ORCID iDs will not appear in the published article."*

**Response:** All co-authors have been informed and will verify their ORCID iDs within the submission system prior to final acceptance.

**C.2: Figure Technical Requirements**

**Editor’s note:** "To ensure your figures meet our technical requirements, please review our figure guidelines at https://journals.plos.org/plosone/s/figures."

**Response:** We have reviewed the PLOS ONE figure guidelines and revised both figures accordingly. All figure files have been converted to TIFF format with LZW compression at 600 dpi resolution, saved in RGB colour mode, and renamed to comply with PLOS ONE file naming conventions (Fig1.tif, Fig2.tif). Figure dimensions fall within the specified range, and all text within figures uses standard fonts at 8–12 point size. The revised figure files have been uploaded as individual files separate from the manuscript, with captions placed in the manuscript text immediately following the paragraph where each figure is first cited.

We hope that the above revisions adequately address the concerns raised by the Academic Editor and the Reviewer. We believe the revised manuscript has been substantially strengthened and would be grateful for the opportunity to have it reconsidered for publication in PLOS ONE.

Yours sincerely,

**Assoc. Prof. Salim Yılmaz**

*(On behalf of all authors)*
