# Supplementary material for: Multisystem frailty phenotypes and associated factors among older adults in Türkiye: A nationally representative study
Source: PLoS One. 2026 Jun 26;21(6):e0352767. doi: 10.1371/journal.pone.0352767 (PMC13308790; doi:10.1371/journal.pone.0352767)
Supplement: Peer review history

# Peer review: Decision Letter 0

**DOI:** 10.1371/journal.pone.0352767.r001

**Authors:** Mario Ulises Pérez-Zepeda

1 May 2026

PONE-D-26-08134

Multisystem frailty phenotypes and associated factors among older adults in Türkiye: a nationally representative study

PLOS One

Dear Dr. Yılmaz,

Thank you for submitting your manuscript to PLOS ONE. After careful consideration, we feel that it has merit but does not fully meet PLOS ONE’s publication criteria as it currently stands. Therefore, we invite you to submit a revised version of the manuscript that addresses the points raised during the review process.

Please submit your revised manuscript by Jun 15 2026 11:59PM. If you will need more time than this to complete your revisions, please reply to this message or contact the journal office at plosone@plos.org. When you're ready to submit your revision, log on to https://www.editorialmanager.com/pone/ and select the 'Submissions Needing Revision' folder to locate your manuscript file.

Please include the following items when submitting your revised manuscript:

- A letter that responds to each point raised by the academic editor and reviewer(s). You should upload this letter as a separate file labeled 'Response to Reviewers'.
- A marked-up copy of your manuscript that highlights changes made to the original version. You should upload this as a separate file labeled 'Revised Manuscript with Track Changes'.
- An unmarked version of your revised paper without tracked changes. You should upload this as a separate file labeled 'Manuscript'.

If you would like to make changes to your financial disclosure, please include your updated statement in your cover letter. Guidelines for resubmitting your figure files are available below the reviewer comments at the end of this letter.

If applicable, we recommend that you deposit your laboratory protocols in protocols.io to enhance the reproducibility of your results. Protocols.io assigns your protocol its own identifier (DOI) so that it can be cited independently in the future. For instructions see: https://journals.plos.org/plosone/s/submission-guidelines#loc-laboratory-protocols. Additionally, PLOS ONE offers an option for publishing peer-reviewed Lab Protocol articles, which describe protocols hosted on protocols.io. Read more information on sharing protocols at https://plos.org/protocols?utm_medium=editorial-email&utm_source=authorletters&utm_campaign=protocols.

As the corresponding author, your ORCID iD is verified in the submission system and will appear in the published article. PLOS supports the use of ORCID, and we encourage all coauthors to register for an ORCID iD and use it as well. Please encourage your coauthors to verify their ORCID iD within the submission system before final acceptance, as unverified ORCID iDs will not appear in the published article. *Only*  the individual author can complete the verification step; PLOS staff *cannot*  verify ORCID iDs on behalf of authors.

We look forward to receiving your revised manuscript.

Kind regards,

Mario Ulises Pérez-Zepeda, M.D., Ph.D.

Academic Editor

PLOS One

Journal Requirements:

When submitting your revision, we need you to address these additional requirements.

1. Please ensure that your manuscript meets PLOS ONE's style requirements, including those for file naming. The PLOS ONE style templates can be found at

https://journals.plos.org/plosone/s/file?id=wjVg/PLOSOne_formatting_sample_main_body.pdf and

https://journals.plos.org/plosone/s/file?id=ba62/PLOSOne_formatting_sample_title_authors_affiliations.pdf

2. We note that the grant information you provided in the ‘Funding Information’ and ‘Financial Disclosure’ sections do not match.

When you resubmit, please ensure that you provide the correct grant numbers for the awards you received for your study in the ‘Funding Information’ section.

3. In the online submission form, you indicated that [Data cannot be shared publicly because they are owned by the Turkish Statistical Institute (TURKSTAT). The deidentified survey microdata are available from TURKSTAT upon reasonable request and approval of a formal data access application (contact: https://www.tuik.gov.tr). The authors are not permitted to share the dataset directly under the terms of the data-use agreement. The analytic code used in this study is available from the corresponding author upon reasonable request.].

All PLOS journals now require all data underlying the findings described in their manuscript to be freely available to other researchers, either 1. In a public repository, 2. Within the manuscript itself, or 3. Uploaded as supplementary information.

This policy applies to all data except where public deposition would breach compliance with the protocol approved by your research ethics board. If your data cannot be made publicly available for ethical or legal reasons (e.g., public availability would compromise patient privacy), please explain your reasons on resubmission and your exemption request will be escalated for approval.

4. If the reviewer comments include a recommendation to cite specific previously published works, please review and evaluate these publications to determine whether they are relevant and should be cited. There is no requirement to cite these works unless the editor has indicated otherwise.

[Note: HTML markup is below. Please do not edit.]

Reviewers' comments:

Reviewer's Responses to Questions

**Comments to the Author**

1. Is the manuscript technically sound, and do the data support the conclusions?

The manuscript must describe a technically sound piece of scientific research with data that supports the conclusions. Experiments must have been conducted rigorously, with appropriate controls, replication, and sample sizes. The conclusions must be drawn appropriately based on the data presented.

Reviewer #1: Yes

**********

2. Has the statistical analysis been performed appropriately and rigorously?

Reviewer #1: Yes

**********

3. Have the authors made all data underlying the findings in their manuscript fully available?

The PLOS Data policy requires authors to make all data underlying the findings described in their manuscript fully available without restriction, with rare exception (please refer to the Data Availability Statement in the manuscript PDF file). The data should be provided as part of the manuscript or its supporting information, or deposited to a public repository. For example, in addition to summary statistics, the data points behind means, medians and variance measures should be available. If there are restrictions on publicly sharing data—e.g. participant privacy or use of data from a third party—those must be specified.

Reviewer #1: Yes

**********

4. Is the manuscript presented in an intelligible fashion and written in standard English?

PLOS ONE does not copyedit accepted manuscripts, so the language in submitted articles must be clear, correct, and unambiguous. Any typographical or grammatical errors should be corrected at revision, so please note any specific errors here.

Reviewer #1: Yes

**********

5. Review Comments to the Author

Please use the space provided to explain your answers to the questions above. You may also include additional comments for the author, including concerns about dual publication, research ethics, or publication ethics. (Please upload your review as an attachment if it exceeds 20,000 characters)

Reviewer #1: Using TURKSTAT data, the authors examined the prevalence of frailty and its associated factors among 26,905 older adults, representing approximately 20.2 million adults in Turkey, using a multidimensional index. According to this nationally representative analysis, 48.6% of adults aged 50 and older in Turkey exhibited some degree of frailty; this prevalence is significantly higher than estimates reported in many high-income countries and in previous studies conducted within Turkey. It is also noteworthy that the frailty profiles identified in this study spanned the domains of mobility, sensory function, cognition, and functional status. However, this paper has the following limitations.

The reviewer believes it is necessary to conduct a comparative analysis to determine how the prevalence of frailty changes when widely used standard criteria—such as Fried’s phenotypic criteria or Rockwood’s cumulative deficit criteria—are applied to the study population. The reviewer believes that this analysis will be particularly significant if it leads to the identification of populations previously slipped by conventional criteria or of phenotypes that could not be measured previously.

Based on the findings of this study, the reviewer believes it is necessary to discuss potential preventive and improvement strategies for frailty in the future.

**********

6. PLOS authors have the option to publish the peer review history of their article (what does this mean?). If published, this will include your full peer review and any attached files.

If you choose “no”, your identity will remain anonymous but your review may still be made public.

**Do you want your identity to be public for this peer review?**  For information about this choice, including consent withdrawal, please see our Privacy Policy.

Reviewer #1: **Yes:** Hiroshi Akasaka

**********

[NOTE: If reviewer comments were submitted as an attachment file, they will be attached to this email and accessible via the submission site. Please log into your account, locate the manuscript record, and check for the action link "View Attachments". If this link does not appear, there are no attachment files.]

To ensure your figures meet our technical requirements, please review our figure guidelines: https://journals.plos.org/plosone/s/figures

You may also use PLOS’s free figure tool, NAAS, to help you prepare publication quality figures: https://journals.plos.org/plosone/s/figures#loc-tools-for-figure-preparation.

NAAS will assess whether your figures meet our technical requirements by comparing each figure against our figure specifications.

# Author comment: Author response to Decision Letter 1

**DOI:** 10.1371/journal.pone.0352767.r002

4 May 2026

PART B: Reviewer #1 Comments (Dr. Hiroshi Akasaka)

General remark by Reviewer: "Using TURKSTAT data, the authors examined the prevalence of frailty and its associated factors among 26,905 older adults, representing approximately 20.2 million adults in Turkey, using a multidimensional index. According to this nationally representative analysis, 48.6% of adults aged 50 and older in Turkey exhibited some degree of frailty; this prevalence is significantly higher than estimates reported in many high-income countries and in previous studies conducted within Turkey. It is also noteworthy that the frailty profiles identified in this study spanned the domains of mobility, sensory function, cognition, and functional status. However, this paper has the following limitations."

Response: We thank the reviewer for this positive and accurate summary of our work.

Reviewer Comment 1: Comparative Analysis with Fried/Rockwood Criteria

Reviewer’s comment: "The reviewer believes it is necessary to conduct a comparative analysis to determine how the prevalence of frailty changes when widely used standard criteria—such as Fried’s phenotypic criteria or Rockwood’s cumulative deficit criteria—are applied to the study population. The reviewer believes that this analysis will be particularly significant if it leads to the identification of populations previously slipped by conventional criteria or of phenotypes that could not be measured previously."

Response: We thank the reviewer for this important suggestion. To address this point, we conducted a comparative analysis by constructing two conventional frailty classifications from the available survey data and comparing them with the LPA-derived multisystem profiles.

A modified Fried phenotype was operationalised as a three-item proxy using the closest available approximations to the original criteria: substantial walking difficulty (Washington Group score ≥2) as a proxy for slowness, substantial gripping difficulty (score ≥2) as a proxy for weakness, and no leisure-time physical activity as a proxy for low activity. Two of the five original Fried criteria — unintentional weight loss and exhaustion — were not available in the survey instrument. A Rockwood-style Frailty Index was constructed using a deficit accumulation approach incorporating 11 health deficits rescaled to a 0–1 range, with standard cutpoints applied.

The results revealed notable differences in case identification across approaches. The modified Fried phenotype yielded a similar overall frailty prevalence (49.2%) to the LPA multisystem approach (48.6%), whereas the Frailty Index produced a substantially higher estimate (87.9%). Cross-classification of LPA profiles against the modified Fried phenotype demonstrated high concordance for the Severe profile (93.2% classified as Fried frail) but substantial discordance for the Intermediate profile, in which 47.9% of individuals (n = 4,441; 16.5% of the total sample) were classified as robust by the modified Fried criteria despite exhibiting multisystem impairment. These discordant individuals were characterised by sensory and cognitive vulnerability (mean vision difficulty 0.59; 42.1% memory complaints) with motor impairment below the Fried threshold, confirming that physical phenotype-based criteria systematically overlook individuals whose vulnerability manifests primarily in non-motor domains. Only 96 individuals (0.4%) showed the reverse pattern (Fried frail, LPA robust), indicating that the multisystem approach captured nearly all individuals identified by the physical phenotype plus a substantial additional subpopulation. Cohen's kappa between LPA and the modified Fried phenotype was 0.400, consistent with prior evidence of moderate agreement across frailty instruments.

These findings have been incorporated into the revised manuscript as follows: the methodology is described in the Statistical analysis subsection (Methods), the key results are reported in a new paragraph in the Results section, a detailed discussion contextualising these findings within the broader frailty classification literature has been added to the Discussion section (with supporting references from Xue et al. 2020, Oviedo-Briones et al. 2021, and Van der Elst et al. 2019), and the full cross-classification tables and domain-specific impairment profiles are presented in a new Supplementary Section 11 (Table S13). We believe this analysis directly addresses the reviewer's concern by demonstrating both how frailty prevalence changes under different classification approaches and, importantly, by identifying the specific subpopulation — characterised by sensory-cognitive vulnerability — that is captured by the multisystem approach but missed by conventional physical phenotype criteria.

Reviewer Comment 2: Preventive and Improvement Strategies for Frailty

Reviewer’s comment: "Based on the findings of this study, the reviewer believes it is necessary to discuss potential preventive and improvement strategies for frailty in the future."

Response: We thank the reviewer for highlighting this important aspect. The Discussion section has been substantially expanded to include a more detailed and concrete discussion of preventive and improvement strategies. Specifically, two new paragraphs have been added (replacing the previous brief mention) that address the following points.

First, building on the finding that frailty is increasingly recognised as a potentially reversible condition, we discuss how the identification of sensory-cognitive vulnerability as a distinguishing feature of the intermediate frailty profile has direct implications for screening design. We argue that screening strategies focused exclusively on physical function—as emphasised in traditional Fried-based approaches—risk overlooking a sizeable at-risk subpopulation, and that effective early detection requires multidomain assessment extending beyond motor performance to include routine vision and hearing evaluation, cognitive monitoring, and enquiry into subjective memory complaints.

Second, we discuss specific intervention strategies, including the growing evidence supporting multicomponent programmes that combine physical exercise with cognitive training, nutritional counselling, and social engagement. We highlight the strong protective association observed for daily physical activity in this study (OR 0.49 for severe frailty) as reinforcing the potential of community-based exercise initiatives as a scalable, low-cost preventive strategy. For Türkiye specifically, we discuss the potential for embedding multidomain frailty screening within the existing primary care infrastructure, including family health centres and home-based care programmes. We also note that the pronounced sex and age gradients in frailty prevalence suggest that prevention efforts should prioritise women and adults approaching the 65-year threshold. Finally, we discuss the broader applicability of combining self-reported, questionnaire-based indicators with data-driven profiling methods such as LPA as a practical pathway for population-level frailty surveillance in settings where performance-based clinical measures are not routinely available.

Attachment Submitted filename: Revision Notes.docx

# Peer review: Decision Letter 1

**DOI:** 10.1371/journal.pone.0352767.r003

**Authors:** Mario Ulises Pérez-Zepeda

16 Jun 2026

Multisystem frailty phenotypes and associated factors among older adults in Türkiye: a nationally representative study

PONE-D-26-08134R1

Dear Dr. Yılmaz,

We’re pleased to inform you that your manuscript has been judged scientifically suitable for publication and will be formally accepted for publication once it meets all outstanding technical requirements.

Within one week, you’ll receive an e-mail detailing the required amendments. When these have been addressed, you’ll receive a formal acceptance letter and your manuscript will be scheduled for publication.

An invoice will be generated when your article is formally accepted. Please note, if your institution has a publishing partnership with PLOS and your article meets the relevant criteria, all or part of your publication costs will be covered. Please make sure your user information is up-to-date by logging into Editorial Manager at Editorial Manager® and clicking the ‘Update My Information' link at the top of the page. For questions related to billing, please contact billing support.

If your institution or institutions have a press office, please notify them about your upcoming paper to help maximize its impact. If they’ll be preparing press materials, please inform our press team as soon as possible -- no later than 48 hours after receiving the formal acceptance. Your manuscript will remain under strict press embargo until 2 pm Eastern Time on the date of publication. For more information, please contact onepress@plos.org.

Kind regards,

Mario Ulises Pérez-Zepeda, M.D., Ph.D.

Academic Editor

PLOS One

Additional Editor Comments (optional):

Reviewers' comments:

Reviewer's Responses to Questions

**Comments to the Author**

1. If the authors have adequately addressed your comments raised in a previous round of review and you feel that this manuscript is now acceptable for publication, you may indicate that here to bypass the “Comments to the Author” section, enter your conflict of interest statement in the “Confidential to Editor” section, and submit your "Accept" recommendation.

Reviewer #1: All comments have been addressed

**********

2. Is the manuscript technically sound, and do the data support the conclusions?

The manuscript must describe a technically sound piece of scientific research with data that supports the conclusions. Experiments must have been conducted rigorously, with appropriate controls, replication, and sample sizes. The conclusions must be drawn appropriately based on the data presented.

Reviewer #1: Yes

**********

3. Has the statistical analysis been performed appropriately and rigorously?

Reviewer #1: Yes

**********

4. Have the authors made all data underlying the findings in their manuscript fully available?

The PLOS Data policy requires authors to make all data underlying the findings described in their manuscript fully available without restriction, with rare exception (please refer to the Data Availability Statement in the manuscript PDF file). The data should be provided as part of the manuscript or its supporting information, or deposited to a public repository. For example, in addition to summary statistics, the data points behind means, medians and variance measures should be available. If there are restrictions on publicly sharing data—e.g. participant privacy or use of data from a third party—those must be specified.

Reviewer #1: Yes

**********

5. Is the manuscript presented in an intelligible fashion and written in standard English?

PLOS ONE does not copyedit accepted manuscripts, so the language in submitted articles must be clear, correct, and unambiguous. Any typographical or grammatical errors should be corrected at revision, so please note any specific errors here.

Reviewer #1: Yes

**********

6. Review Comments to the Author

Please use the space provided to explain your answers to the questions above. You may also include additional comments for the author, including concerns about dual publication, research ethics, or publication ethics. (Please upload your review as an attachment if it exceeds 20,000 characters)

Reviewer #1: The authors revised the manuscript in response to each comment the reviewer pointed out, and the revised version is satisfactory. Therefore, the reviewer thinks that the revised version is now suitable for publication.

**********

7. PLOS authors have the option to publish the peer review history of their article (what does this mean?). If published, this will include your full peer review and any attached files.

If you choose “no”, your identity will remain anonymous but your review may still be made public.

**Do you want your identity to be public for this peer review?**  For information about this choice, including consent withdrawal, please see our Privacy Policy.

Reviewer #1: No

**********

# Editor report: Acceptance letter

**DOI:** 10.1371/journal.pone.0352767.r004

**Authors:** Mario Ulises Pérez-Zepeda

PONE-D-26-08134R1

PLOS One

Dear Dr. Yılmaz,

I'm pleased to inform you that your manuscript has been deemed suitable for publication in PLOS One. Congratulations! Your manuscript is now being handed over to our production team.

At this stage, our production department will prepare your paper for publication. This includes ensuring the following:

* All references, tables, and figures are properly cited

* All relevant supporting information is included in the manuscript submission,

* There are no issues that prevent the paper from being properly typeset

You will receive further instructions from the production team, including instructions on how to review your proof when it is ready. Please keep in mind that we are working through a large volume of accepted articles, so please give us a few days to review your paper and let you know the next and final steps.

Lastly, if your institution or institutions have a press office, please let them know about your upcoming paper now to help maximize its impact. If they'll be preparing press materials, please inform our press team within the next 48 hours. Your manuscript will remain under strict press embargo until 2 pm Eastern Time on the date of publication. For more information, please contact onepress@plos.org.

You will receive an invoice from PLOS for your publication fee after your manuscript has reached the completed accept phase. If you receive an email requesting payment before acceptance or for any other service, this may be a phishing scheme. Learn how to identify phishing emails and protect your accounts at https://explore.plos.org/phishing.

If we can help with anything else, please email us at customercare@plos.org.

Thank you for submitting your work to PLOS ONE and supporting open access.

Kind regards,

PLOS ONE Editorial Office Staff

on behalf of

Dr. Mario Ulises Pérez-Zepeda

Academic Editor

PLOS One
